# Real-Time Multifrequency MR Elastography of the Human Brain Reveals Rapid Changes in Viscoelasticity in Response to the Valsalva Maneuver

**DOI:** 10.3389/fbioe.2021.666456

**Published:** 2021-05-05

**Authors:** Helge Herthum, Mehrgan Shahryari, Heiko Tzschätzsch, Felix Schrank, Carsten Warmuth, Steffen Görner, Stefan Hetzer, Hennes Neubauer, Josef Pfeuffer, Jürgen Braun, Ingolf Sack

**Affiliations:** ^1^Institute of Medical Informatics, Charité – Universitätsmedizin Berlin, Corporate Member of Freie Universität Berlin, Humboldt-Universität zu Berlin, and Berlin Institute of Health, Berlin, Germany; ^2^Department of Radiology, Charité – Universitätsmedizin Berlin, Corporate Member of Freie Universität Berlin, Humboldt-Universität zu Berlin, and Berlin Institute of Health, Berlin, Germany; ^3^Berlin Center for Advanced Neuroimaging (BCAN), Berlin, Germany; ^4^Application Development, Siemens Healthcare GmbH, Erlangen, Germany

**Keywords:** real-time multifrequency MRE, cerebral autoregulation, Valsalva maneuver, stiffness, viscoelasticity

## Abstract

Modulation of cerebral blood flow and vascular compliance plays an important role in the regulation of intracranial pressure (ICP) and also influences the viscoelastic properties of brain tissue. Therefore, magnetic resonance elastography (MRE), the gold standard for measuring *in vivo* viscoelasticity of brain tissue, is potentially sensitive to cerebral autoregulation. In this study, we developed a multifrequency MMRE technique that provides serial maps of viscoelasticity at a frame rate of nearly 6 Hz without gating, i.e., in quasi-real time (rt-MMRE). This novel method was used to monitor rapid changes in the viscoelastic properties of the brains of 17 volunteers performing the Valsalva maneuver (VM). rt-MMRE continuously sampled externally induced vibrations comprising three frequencies of 30.03, 30.91, and 31.8 Hz were over 90 s using a steady-state, spiral-readout gradient-echo sequence. Data were processed by multifrequency dual elasto-visco (MDEV) inversion to generate maps of magnitude shear modulus | G^∗^| (stiffness) and loss angle φ at a frame rate of 5.4 Hz. As controls, the volunteers were examined to study the effects of breath-hold following deep inspiration and breath-hold following expiration. We observed that | G^∗^| increased while φ decreased due to VM and, less markedly, due to breath-hold in inspiration. Group mean VM values showed an early overshoot of | G^∗^| 2.4 ± 1.2 s after the onset of the maneuver with peak values of 6.7 ± 4.1% above baseline, followed by a continuous increase in stiffness during VM. A second overshoot of | G^∗^| occurred 5.5 ± 2.0 s after the end of VM with peak values of 7.4 ± 2.8% above baseline, followed by 25-s sustained recovery until the end of image acquisition. φ was constantly reduced by approximately 2% during the entire VM without noticeable peak values. This is the first report of viscoelasticity changes in brain tissue induced by physiological maneuvers known to alter ICP and detected by clinically applicable rt-MMRE. Our results show that apnea and VM slightly alter brain properties toward a more rigid-solid behavior. Overshooting stiffening reactions seconds after onset and end of VM reveal rapid autoregulatory processes of brain tissue viscoelasticity.

## Introduction

A balance of intracranial mechanical properties is of crucial importance for normal brain function (Linninger et al., 2005; Guyton and Hall, 2006; Wagshul et al., 2006; Schmid Daners et al., 2012). Shear modulus and bulk modulus of brain tissue influence cerebrovascular compliance and pulsatility as well as intracranial pressure (ICP) (Giulioni et al., 1988; Greitz et al., 1992; Wagshul et al., 2011; Parker, 2017). While shear modulus can be measured non-invasively by magnetic resonance elastography (MRE) (Hirsch et al., 2017), there is currently no method for direct ICP measurement without an intervention or without making model assumptions (Guyton and Hall, 2006). In complex multiphasic mechanical systems such as the brain, shear modulus and pressure are linked through poroelastic interactions between the fluid and solid spaces (Bilston, 2002; Tully and Ventikos, 2011; Parker, 2014). Thus, it is likely that regulation of ICP, which is one of the most important vital functions of intracranial mechanics, also affects shear viscoelasticity (Perrinez et al., 2009; McGarry et al., 2015; Lilaj et al., 2020). However, this mechanical component of cerebral autoregulation is largely unstudied due to a lack of imaging techniques that can measure cerebral shear modulus *in vivo* with high spatial and temporal resolution.

In the past, cerebral MRE was used to study a wide variety of physiological effects or diseases which affect the *in vivo* shear modulus of brain tissue (Hiscox et al., 2016; Yin et al., 2018). It has been shown that the brain becomes softer during normal aging (Sack et al., 2009; Arani et al., 2015) or pathophysiological processes such as neuroinflammation (Riek et al., 2012; Wang et al., 2019), demyelination (Schregel et al., 2012), or neurodegeneration (Murphy et al., 2012; Munder et al., 2018). In patients, brain softening has been observed in a wide set of neuronal disorders including multiple sclerosis (Wuerfel et al., 2010; Fehlner et al., 2016), Alzheimer’s disease (Murphy et al., 2011; Murphy et al., 2016; Gerischer et al., 2018), Parkinson’s disease (Lipp et al., 2013, 2018) and normal pressure hydrocephalus (Streitberger et al., 2011; Juge et al., 2016; Murphy et al., 2020). Brain tumors can be either softer or stiffer than normal tissue (Simon et al., 2013; Jamin et al., 2015; Reiss-Zimmermann et al., 2015), while malignant tumors have reduced viscosity (Streitberger et al., 2014; Schregel et al., 2018; Streitberger et al., 2020). A higher stiffness of neural tissue has been associated with increased perfusion pressure (Chatelin et al., 2015; Hetzer et al., 2018, 2019; Bertalan et al., 2019a), ICP (Hatt et al., 2015; Arani et al., 2018), formation of cytotoxic edema in dying animals (Weickenmeier et al., 2018; Bertalan et al., 2020), proliferation of neurons (Klein et al., 2014), neuronal activity (Patz et al., 2019; Lan et al., 2020), and brain maturation (Guo et al., 2019). All of these studies have revealed that brain viscoelasticity can change within minutes (perfusion alterations), weeks (brain maturation in mice), or years (aging, disease progression). However, requiring several minutes of data acquisition, conventional MRE is limited in resolving non-periodic rapid processes such as cerebral autoregulation and ICP alterations which cannot be consistently repeated in volunteers.

Faster techniques including time-harmonic ultrasound elastography (Tzschatzsch et al., 2018; Kreft et al., 2020) and real-time MRE (rt-MRE) (Schrank et al., 2020a) have been introduced recently. While cerebral ultrasound elastography is limited by acoustic windows and cannot generate detailed maps, rt-MRE has the potential to map viscoelasticity with both high spatial resolution and high frame rates. However, feasibility of rt-MRE has as yet only been demonstrated with a small field of view in the lower extremities (Schrank et al., 2020a) and has never been tested in the brain.

Therefore, we here introduce real-time multifrequency MRE (rt-MMRE) for applications in the human brain. Multifrequency extension of rt-MRE was motivated by previous work on multifrequency wavefield inversion promising higher stability and consistency of parameter maps than single-frequency direct inversion (Papazoglou et al., 2012; Hirsch et al., 2014). Moreover, rt-MRE builds on continuous stroboscopic sampling of harmonic vibrations (Schrank et al., 2020b), which can be spectrally decomposed into multifrequency vibrations without extra scan time. As such, rt-MMRE is a natural extension of rt-MRE that yields, at no extra cost, consistent viscoelasticity maps at relatively high frame rates in the order of 6 Hz depending on the repetition time (TR). Since rt-MRE does not require gating and provides multiple viscoelasticity maps per second, we consider this method as a real-time imaging technique.

Using rt-MMRE, we investigate rapid viscoelastic changes during cerebral autoregulation associated with the Valsalva maneuver (VM). The VM is a standard maneuver to voluntarily increase ICP by forceful breathing against the closed airway with abdominal muscle contraction at the same time. VM will be compared with normal breath-holds in inspiration (BH-in) and expiration (BH-ex). To address frequency dispersion and to test the overall consistency of the values measured in association with the VM, the experiment is repeated with a second set of drive frequencies.

Overall, this study has two aims: first, we introduce rt-MMRE based on three simultaneous excitation frequencies to acquire hundreds of viscoelasticity maps within less than 1 min of scan time. Second, we explore cerebral autoregulation with the unprecedentedly high spatiotemporal resolution offered by rt-MMRE.

## Materials and Methods

### Subjects

rt-MMRE was performed in 17 healthy volunteers without a history of neurological diseases (5 females, 36 ± 13 years, age range: 25–81 years, randomly selected). The study was approved by the ethics committee of Charité – Universitätsmedizin Berlin in accordance with the Ethical Principles for Medical Research Involving Human Subjects of the World Medical Association Declaration of Helsinki. Every participant gave written informed consent. Participant characteristics are summarized in Table 1. Group mean time curves of heart rate are given in Inline Supplementary Figure 1.

**TABLE 1:** Participant characteristics with abbreviations: body mass index (BMI), systolic blood pressure (BPsys), diastolic blood pressure (BPdis), and heart rate (HR).

**ID**	**Sex**	**Age in years**	**BMI in kg/m^2^**	**BPsys in mmHg**	**BPdia in mmHg**	**HR in bpm**
1	f	37	17.5	97	56	69
2	m	29	24.2	130	77	80
3	m	43	23.6	150	88	88
4	m	46	26.3	134	85	62
5	m	34	22.7	124	68	76
6	m	25	20.8	120	70	70
7	m	27	21.6	118	70	64
8	m	30	26.3	126	78	78
9	f	28	20.7	131	85	62
10	m	36	19.9	77	50	54
11	m	26	20.2	121	75	80
12	f	26	20.5	113	60	55
13	f	29	25.7	114	72	73
14	m	51	20.7	130	85	62
15	m	37	26.2	122	72	70
16	f	27	31.6	140	78	90
17	m	81	22.5	125	80	70
Mean (SD)	–	36 (13)	23 (3)	122 (16)	74 (10)	71 (10)

### Experimental Setup

**FIGURE 1:**
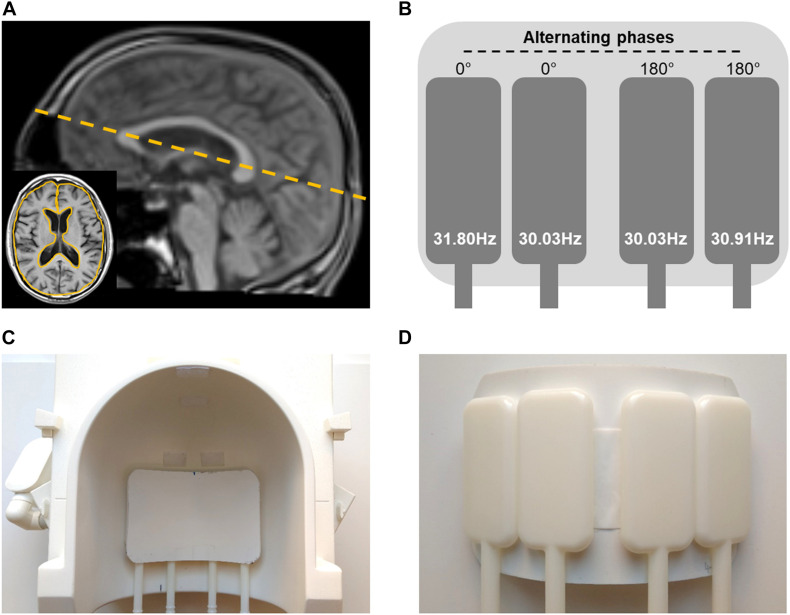
Experimental setup. **(A)** Image slice position (dashed yellow line) and region of interest (ROI) based on anatomical image (yellow solid line in the insert) for rt-MMRE of the brain. **(B)** Diagram of the four flask drivers with vibration frequencies and 180°-phase alterations between the drivers. **(C)** Top view of positioning of actuator setup in the 32-channel head coil. **(D)** Bottom view of driver setup.

All experiments were performed in a 3T MRI scanner (Siemens MAGNETOM Prisma, Erlangen) using a 32-channel head coil. Triple-harmonic vibrations in a narrowband frequency regimen were synchronously induced by four pressurized air drivers attached to a transmission plate and placed underneath the head. The applied frequencies were: 30.03, 30.91, and 31.8 Hz (hereinafter referred to as 31-Hz regimen). The two outmost drivers were operated at the highest frequencies with alternated phases relative to each other. The two inner drivers were operated with the same frequency, again with alternated phases. This way each frequency induced mainly lateral-rotational head motion with minimized compression components. The setup is shown in Figure 1.

Vibrations and radiofrequency (RF) excitation started 5 s before data acquisition to ensure establishment of steady states of time-harmonic oscillations and magnetization before start of each experiment. The following rt-MMRE experiments were performed:

i Valsalva maneuver (VM)
ii deep inspiration and breath-hold (BH-in)
iii expiration and breath-hold (BH-ex)

The VM experiment included four consecutive phases: 30 s baseline, 5 s breath-hold in inspiration, 20 s VM and 35 s recovery (total scan time: 90 s). Prior to the experiment, subjects were trained to perform a moderate Valsalva maneuver that could be easily sustained for 20 s to prevent involuntary movement after deep breathing. This experiment was repeated with a second narrowband frequency regimen comprising 40.77, 41.67, and 42.55 Hz (hereinafter referred to as 42-Hz regimen) in order to check the overall consistency of MRE during VM and if there is a noticeable influence of frequency.

BH-in and BH-ex experiments consisted of 30-s baseline acquisition with the volunteer breathing normally, followed by a 25-s breath-hold in inspiration or expiration, and a final 35 s recovery phase (total scan time: 90 s).

A resting period of at least 30 s was observed between the experiments. Start and stop commands were given as visual signals to the volunteers. The finger pulse was continuously recorded to track changes in heart rate.

Additionally, anatomical images were acquired using a T1-weighted, turbo-spin echo (TSE) sequence.

### rt-MMRE Pulse Sequence

**FIGURE 2:**
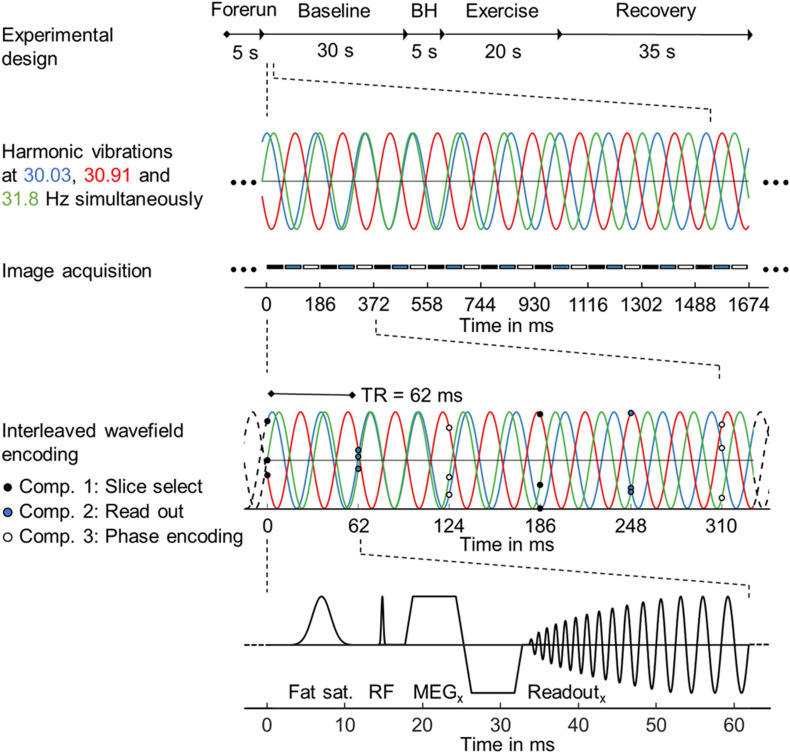
Steady-state gradient echo timing diagram with spiral readout trajectory for single-shot multifrequency real-time MRE. From top to bottom: Experimental design with timing, harmonic vibrations at three frequencies over a period of 9 × 3 TRs with stroboscopic image acquisition below, interleaved wavefield encoding over 2 × 3 TRs, simplified sequence diagram with combined RF and x-gradients to illustrate fat saturation, RF excitation, motion encoding and spiral readout by x-gradients over a single TR period.

Single-frequency rt-MRE using a 2D single-shot gradient echo MRE pulse sequence with spiral readout was recently introduced for directly mapping skeletal muscle function (Schrank et al., 2020a). For rt-MMRE we used a similar prototype—a single-shot, gradient-echo sequence with dual-density spiral readout, which samples multifrequency vibrations in a stroboscopic fashion as illustrated in Figure 2. TR was 62 ms including RF excitation with 20° flip angle, 20 ms TE, 28 ms readout length, signal spoiling and fat suppression. For motion encoding, a single-cycle, bipolar motion-encoding gradient of 17.5-ms duration (57 Hz) and 40-mT/m amplitude was deployed within each TR according to the principle of fractional encoding (Rump et al., 2007). Images were reconstructed using the SPIRiT non-Cartesian parallel imaging technique (Lustig and Pauly, 2010). Three Cartesian motion components were encoded in an interleaved fashion within the series of consecutive TRs, yielding a sequence of 1,458 wave images. Collapsing these three components into a single viscoelasticity map resulted in a total MRE frame rate of 3 × TR = 186 ms, or approximately 5.4 Hz.

Data were acquired in a single transverse image slice with a field of view (FoV) of 192 × 192 mm^2^ and 2 × 2 × 5 mm^3^ voxel size. The slice was automatically positioned using the localizer-based auto-align function at the level of the basal nuclei along the largest diameter of the lateral ventricles in the sagittal plane as shown in Figure 1.

### Parameter Reconstruction

**FIGURE 3:**
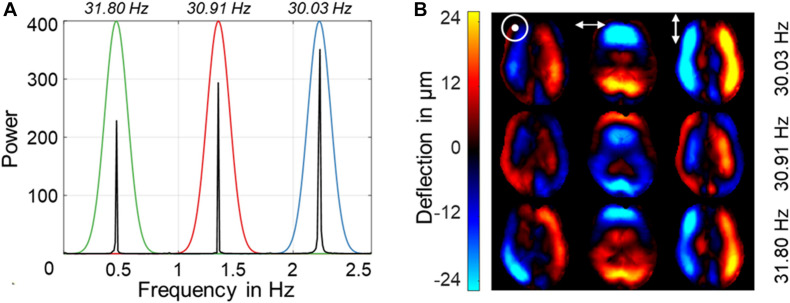
Representative Fourier power spectra with three aliased excitation frequencies for one motion-encoding component above and wave deflections for three encoding components and three vibration frequencies below. **(A)** Power spectra for vibrations at 31-Hz regimen. Color coding indicates the respective vibration frequency with Gaussian bandpass filter used for Hilbert transformation. The frequency axis is scaled from 0 to the Nyquist frequency in Hz, which is determined by the sampling rate of 5.4 Hz. Stroboscopic sampling of multiharmonic vibrations causes all frequencies to be aliased within this limited frequency window. **(B)** Representative wave images after frequency decomposition for the three encoding components using the 31-Hz regimen (30.03, 30.91, 31.8 Hz). [ʘ,↔,↕ denote deflections through-plane (head-to-feet), left-right, and up-down (anterior-posterior), respectively].

The 1,458 raw, complex-valued MR images were smoothed with a Gaussian filter (σ = 0.65 px) and subsequently unwrapped using gradient unwrapping (Hirsch et al., 2017). The three vibration frequencies were decomposed by temporal Fourier transformation. Due to stroboscopic undersampling of vibrations in rt-MMRE, the frequencies appeared at aliased positions in the spectrum (see Figure 3A). The frequencies were selected by three Gaussian bandpass filters (σ = 0.1 Hz) each of which centered at the expected (aliased) frequency of the fundamental drive frequency. These filters were used for inverse Hilbert transformation to compute complex-valued wave fields (wave images) for each vibration frequency, separately yielding 4,374 (1,458 × 3 vibration frequencies) time-resolved wave images (Schrank et al., 2020b). Nine wave images of three Cartesian field components and three vibration frequencies (see Figure 3B) were fed into multifrequency dual elasto-visco inversion (Papazoglou et al., 2012), yielding 486 (4,374/3 encoding components/3 vibration frequencies) consecutive maps of stiffness (| G^∗^|) and loss angle (φ) with 5.4-Hz frame rate over the entire examination time. While | G^∗^| is a measure of stiffness, φ describes the ratio of elastic to viscous tissue properties indicating fluid properties as explained in Streitberger et al. (2020) | G^∗^| and φ maps from the beginning and end of the series were discarded within 5-s margins to minimize transient effects introduced by periodic boundary conditions of the Hilbert transform. Consequently, the final observation window was 80 s. All data processing was done in MATLAB (version 2020a). The inversion pipeline is publicly available at https://bioqic$-$apps.charite.de (Meyer et al., 2019). Main results are given in Table 1, Table 2 and in Supplementary Tables 1a–c. Raw data can be made available upon request without restrictions.

**TABLE 2:** Mean | G*| (SD) in Pa and mean φ (SD) in rad for each phase and participant in the Valsalva maneuver experiments using the 31-Hz regimen (30.03, 30.91, 31.8 Hz).

**ID**	Mean | G*| (SD) in Pa				**Mean φ (SD) in rad**			
	**BSL**	**ESM**	**LRM**	**REC**	**BSL**	**ESM**	**LRM**	**REC**
1	1290 (6)	1323 (17)	1313 (5)	1290 2 (8)	0.841 (0.001)	0.831 (0.001)	0.849 (0.001)	0.840 (0.001)
2	1243 (16)	1340 (17)	1327 (10)	1237 (11)	0.816 (0.007)	0.798 (0.003)	0.799 (0.003)	0.817 (0.001)
3	1351 (11)	1415 (14)	1409 (5)	1336 (9)	0.783 (0.001)	0.769 (0.003)	0.791 (0.005)	0.783 (0.002)
4	1490 (12)	1530 (29)	1525 (6)	1495 (8)	0.87 (0.002)	0.853 (0.001)	0.87 (0.003)	0.868 (0.004)
5	1441 (6)	1484 (24)	1566 (14)	1456 (10)	0.799 (0.003)	0.777 (0.005)	0.811 (0.009)	0.794 (0.004)
6	1502 (4)	1536 (7)	1609 (3)	1511 (7)	0.795 (0.001)	0.789 (0.004)	0.799 (0.002)	0.795 (0.002)
7	1321 (8)	1363 (17)	1395 (14)	1301 (11)	0.818 (0.004)	0.815 (0.006)	0.83 (0.002)	0.829 (0.002)
8	1495 (6)	1519 (20)	1655 (8)	1534 (16)	0.791 (0.002)	0.763 (0.004)	0.796 (0.008)	0.79 (0.004)
9	1515 (7)	1620 (9)	1617 (6)	1549 (1)	0.809 (0.003)	0.765 (0.008)	0.778 (0.006)	0.792 (0.001)
10	1395 (4)	1431 (8)	1473 (8)	1401 (8)	0.78 (0.002)	0.776 (0.004)	0.779 (0.003)	0.778 (0.001)
11	1237 (12)	1238 (11)	1312 (15)	1242 (9)	0.807 (0.004)	0.806 (0.003)	0.81 (0.005)	0.804 (0.001)
12	1312 (11)	1350 (7)	1416 (11)	1310 (11)	0.758 (0.003)	0.742 (0.004)	0.755 (0.001)	0.754 (0.003)
13	1509 (4)	1510 (15)	1572 (2)	1509 (5)	0.809 (0.001)	0.807 (0.001)	0.804 (0.001)	0.8 (0.001)
14	1373 (8)	1407 (14)	1392 (3)	1353 (4)	0.792 (0.002)	0.777 (0.002)	0.787 (0.002)	0.786 (0)
15	1295 (8)	1421 (35)	1407 (13)	1325 (15)	0.784 (0.005)	0.741 (0.018)	0.796 (0.002)	0.781 (0.003)
16	1356 (7)	1391 (19)	1422 (2)	1330 (18)	0.813 (0.003)	0.795 (0.004)	0.805 (0.002)	0.805 (0.003)
17	1157 (3)	1192 (9)	1175 (12)	1212 (6)	0.733 (0.003)	0.725 (0.002)	0.722 (0.002)	0.728 (0.001)
Mean (SD)	1370 (106)	1416 (108)	1446 (126)	1376 (108)	0.800 (0.030)	0.784 (0.032)	0.799 (0.032)	0.797 (0.031)

*BSL, Baseline; ESM, established maneuver; LRM, late response maneuver; REC, recovery.*

### Parameter Analysis and Statistical Tests

**FIGURE 4:**
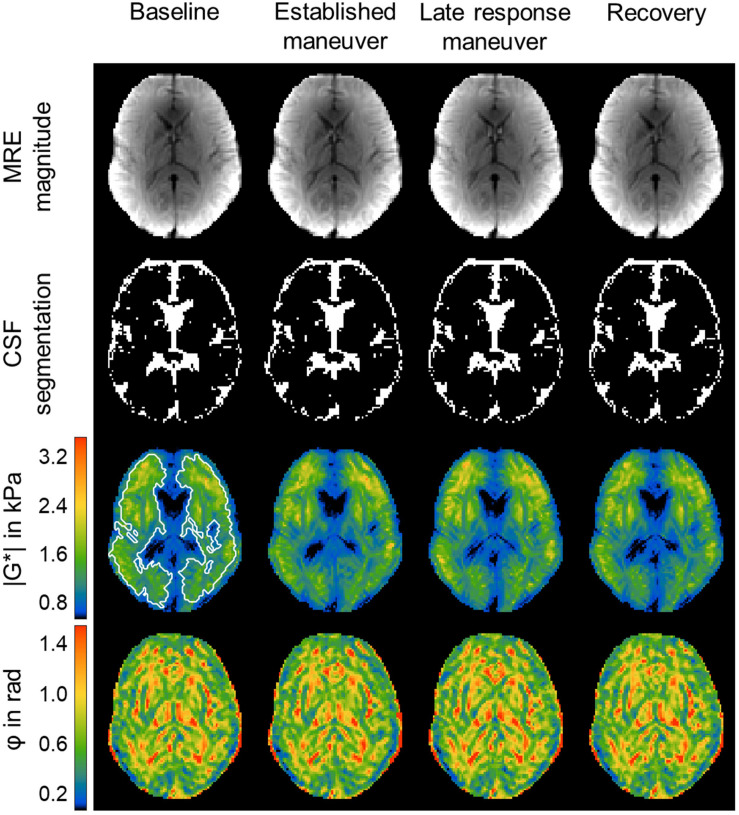
Representative rt-MMRE MRE magnitude, CSF masks, | G*| and φ maps of the *in vivo* human brain. Time-averaged MRE magnitude, derived CSF masks, | G*| and φ maps of one volunteer over the four phases [baseline (BSL), established maneuver (ESM), late response maneuver (LRM) and recovery (REC)] of the VM experiment using the 31-Hz regimen (30.03, 30.91, 31.8 Hz). The number of CSF associated voxels for each phase was BSL: 1079, ESM: 1128, LRM: 1069, REC: 1074. The | G*| maps show slightly elevated values throughout the slice. The region of interest (ROI) is indicated by white lines. The same ROI was used for all phases and for the φ maps as well.

For every time frame, | G^∗^| and φ were quantified by averaging values over the same region of interest (ROI). ROIs were manually drawn based on anatomical T1-weighted images, as shown in Figure 1A. Furthermore, these ROIs were refined by empirical thresholds of 10 (time-averaged MRE signal magnitude) and of 950 Pa (time-averaged | G^∗^| map) to remove ventricles and larger sulci similar to Shahryari et al. (2020) (see Figure 4).

The same ROI was also used to determine magnetization signal-to-noise ratio (SNR) and wave displacement SNR (WSNR) for every time frame. WSNR was derived using the blind noise estimation method proposed by Donoho et al. (1995) as outlined and previously applied to MRE data in Bertalan et al. (2019b) and Schrank et al. (2020a) This noise estimation method is suited for wave image analysis since the spatial frequencies of MRE waves and noise are well separated in the wavelet domain (Selesnick et al., 2005; Barnhill et al., 2017).

To test if multifrequency inversion yields more stable values than single-frequency inversion we determined the coefficient of variation (CV) during the baseline phase prior to VM, BH-in and BH-ex for both | G^∗^| and φ in all volunteers. The same raw data was used, but for the single-frequency inversion only one frequency from the temporal Fourier spectrum was selected.

We further analyzed difference | G^∗^| and φ values relative to mean baseline values given as Δ| G^∗^|=| G^∗^| _(t)_ − | G^∗^| _(baseline__)_ (correspondingly for Δφ) in order to quantify individual parameter changes. In addition, peak viscoelasticity values and their temporal delays relative to the onset and end of VM were identified and tabulated for each volunteer.

Finally, group statistics was applied to the absolute values of | G^∗^| and φ, after temporal averaging over the following experimental phases for each participant:

(1) Baseline (BSL): 2.5–22.5 s
(2) Established maneuver (ESM): 32.5–47.5 s
(3) Late response maneuver (LRM): 52.5–57.5 s
(4) Recovery (REC): 70–80 s.

**FIGURE 5:**
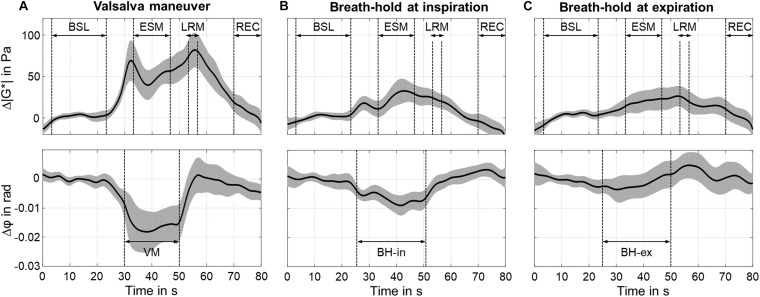
Time courses of group mean values of Δ| G*| (top of subfigures) and Δφ (bottom of subfigures) using the 31-Hz regimen (30.03, 30.91, 31.8 Hz). The gray areas show 95% confidence intervals. For Valsalva maneuver (VM), timing was as follows: breath-hold in inspiration (BH-in) at 25 s, start of VM at 30 s, stop of VM at 50 s. For breath-hold (BH) experiments, timing was as follows: start of BH at 25 s, stop at 50 s. **(A)** Valsalva maneuver (VM). **(B)** Breath-hold in inspiration (BH-in). **(C)** Breath-hold in expiration (BH-ex).

Of note, these time intervals were given by the aforementioned study design (30-25-35 s. for baseline-breathold/VM-recovery) minus 2.5 s transition phases at the beginnings and ends of these phases including an additional late-response phase. The transition phases were discarded from our analysis in order to minimize transients resulted by the frequency bandpass filter. Also, 5 s BH (25–30 s) and 10 s of post-VM (60–70 s) were considered as transition phases and henceforth not included in our group statistical analysis. All phases are demarcated in Figure 5.

To test possible deformations of lateral ventricles due to VM as reported previously (Ertl-Wagner et al., 2001), we applied automatic segmentation of cerebral spinal fluid (CSF) to the temporal averaged MRE magnitude images of the different experimental phases using SPM12 (Penny et al., 2011; see Figure 4). CSF probability maps were thresholded at 0.5 to generate logical CSF-associated voxel masks. A linear mixed-effects model with varying intercept was employed. CSF volume was used as dependent variable and the individual phases as independent variables. Participants were assigned as random effect, and *P*-values were calculated using Tukey’s *post hoc* test with Bonferroni correction for multiple comparisons. To test for significant changes in | G^∗^| and φ between phases (1)–(4), a linear mixed-effects model with varying intercept was employed. | G^∗^| and φ were used as dependent variables and the individual phases as independent variables. Participants were assigned as random effect, and *P*-values were calculated using Tukey’s *post hoc* test with Bonferroni correction for multiple comparisons. This test does not account for inter-individual slope variations of | G^∗^| and φ but analyzes the significance of temporal changes of these parameters. SNR and viscoelastic parameters were correlated using a linear mixed model with | G^∗^| and φ as dependent variables and SNR or WSNR as fixed effects with subjects as random factor. All statistical analysis was done in R (version 3.6.2). Unless otherwise stated, errors are given as standard deviation (SD). Correlations between viscoelastic baseline values as well as individual peak responses and participant characteristics (see Table 1) were analyzed using Pearson’s correlation coefficient. *P*-values below 0.05 were considered statistically significant.

## Results

Variation in baseline | G^∗^| and φ was smaller when using multifrequency inversion (CV = 0.74%, 0.51%) than single frequencies (CV = 0.99%, 0.77%, *P* < 0.001).

Figure 4 shows representative time-averaged MRE magnitude images, automatically segmented CSF masks, as well as | G^∗^| and φ maps acquired during the four phases of the experiment. A slight increase in | G^∗^| was visible in the late VM response, whereas no response of φ was apparent in individual maps. Group statistics revealed no significant change of CSF-associated voxels between the different states of the maneuver. A descriptive statistic for the individual phases of the VM experiment in the 31-Hz regimen and for each participant is given in Supplementary Table 2.

### Relative | G^∗^| and φ Changes

Individual analysis of | G^∗^| showed an increase (6.7 ± 4.1%, *P* < 0.001) at approximately 2.4 ± 1.2 s after start of VM and 5.5 ± 2.0 s after end of VM (7.4 ± 2.8%. *P* < 0.001). φ decreased during ESM (−2.1 ± 1.4%, *P* < 0.001). Averaged time courses of Δ| G^∗^| and Δφ are presented in Figure 5. An early peak of Δ| G^∗^| showed a difference of 69 ± 50 Pa (*P* < 0.001) from baseline values. After a short drop, Δ| G^∗^| steadily increased during ESM. The second overshoot differed from baseline by 82 ± 42 Pa (*P* < 0.01). Δ| G^∗^| recovered toward baseline values once the volunteers returned to normal breathing. Δφ was constantly decreased during ESM (−0.018 ± 0.012 rad, *P* < 0.01).

The BH-in experiment showed an increase in Δ| G^∗^| after 3.0 ± 1.0 s (18 ± 16 Pa, *P* < 0.001) with a maximum at 17.0 ± 2.0 s after start of BH-in (32 ± 29 Pa, *P* < 0.001). Δφ decreased during ESM (−0.006 ± 0.004 rad, *P* < 0.001) reaching a minimum at 17 ± 2 s after start of BH-in (−0.009 ± 0.007 rad, *P* < 0.001).

The BH-ex experiment showed no clear peak, neither in Δ| G^∗^| nor Δφ. | G^∗^| increased continuously with onset of BH-ex and reached a maximum 2.5 ± 1.5 s after the end of BH-ex (26 ± 23 Pa, *P* < 0.001).

### Absolute | G^∗^| and φ Changes

**FIGURE 6:**
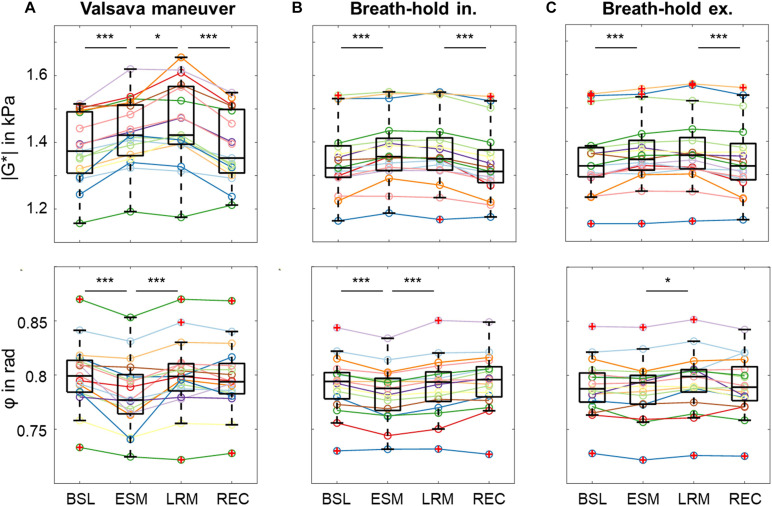
Group values as boxplots for the absolute values of | G*| (top) and φ (bottom) in each phase illustrate the changes in viscoelastic properties induced by the different maneuvers using the 31-Hz regimen for each phase [baseline (BSL), established maneuver (ESM), late response maneuver (LRM), recovery (REC)]. **(A)** Valsalva maneuver (VM). **(B)** Breath-hold in inspiration (BH-in). **(C)** Breath-hold in expiration (BH-ex). Asterisks at the top demarcate significant changes in | G*| and φ which were determined from a linear mixed-effects model with varying intercept. | G*| and φ assigned dependent variables and the individual phases as independent variables. Participants were assigned as random effect, and *P*-values were calculated using Tukey’s *post hoc* test with Bonferroni correction for multiple comparisons. (*P < 0.05, ***P < 0.001).

Figure 6 shows boxplots with median effects for different states of the maneuver for | G^∗^| and φ. The significance levels, indicated by asterisks, were determined from a linear mixed model analysis with varying intercept and participants as random effect. For the VM, different individual effect sizes were observed; however, all subjects showed an increase in | G^∗^| and a decrease in φ due to the maneuver. Averaged | G^∗^| values changed between all phases of the experiment (range: 1,370–1,446 Pa) with significance levels indicated in the figure. Averaged φ values changed both from BSL to ESM and again from ESM to LRM (range: 0.784–0.800 rad).

By contrast, | G^∗^| only changed at the start and end of the maneuver in BH-in (range: 1,338–1,372 Pa), whereas φ changed between BSL and ESM as well as between ESM and LRM (range: 0.783–0.792 rad). In the BH-ex experiment, | G^∗^| changed between LRM and REC (range: 1,348–1,371 Pa) while φ changed between ESM and LRM (range: 0.786–0.791 rad).

Results of the second VM experiment performed using the 42-Hz regimen are presented in Supplementary Material. No significant differences in viscoelastic responses between the 31-Hz and 42-Hz regimen were observed (*P* = 0.24).

Descriptive statistics of | G^∗^| in Pa and φ in rad for the individual phases of the VM experiment and for each participant are summarized in Table 2A. Correspondingly, statistical results for the breath-hold and VM experiments performed with the 42-Hz regimen are presented in Supplementary Tables 1a–c. Participant characteristics did not correlate with | G^∗^| or φ.

### SNR Analysis

Time-averaged SNR and WSNR values did not change significantly across volunteers and over time (*P* = 0.43). Mean SNR was 29 ± 2 dB across all volunteers with minor and insignificant variations of ± 0.5 dB over the course of the experiment. Mean WSNR was 36 ± 2 dB with minor and insignificant variations of ± 1 dB over the course of the experiment. Significant correlation between group mean | G^∗^| and φ was observed (31-Hz regimen: *R* = −0.4, *P* < 0.001, 42-Hz regimen: *R* = −0.5, *P* < 0.001).

## Discussion

This paper presents a novel rt-MMRE technique for the *in vivo* measurement of rapid and non-periodic changes in brain viscoelasticity in humans. MRE exploiting stroboscopic sampling of multifrequency harmonic vibrations revealed the viscoelastic response of brain tissue to the Valsalva maneuver. Overall, the extension of rt-MRE to rt-MMRE by simultaneous excitation of multifrequency oscillations has increased the consistency of our measurements without adding scanning time. Probably for this reason, all subjects consistently showed an increase in | G^∗^| and a decrease in φ with VM, resulting in high statistical significance. This basic finding is remarkable, since the VM is known to induce variability by subjective pressure generation. To further discuss our results we start by briefly reviewing the basic effects of VM on cerebral perfusion and ICP.

### Physiological Effects of VM on Cerebral Blood Flow, ICP and MRE

In this study, elevation of intrathoracic pressure during VM was induced by deep inspiration following and increased abdominal pressure similar to the maneuver used in Ipek-Ugay et al. (2017). With onset of VM and elevated intrathoracic pressure, arterial blood pressure (ABP) increases (Elisberg, 1963; Smith et al., 1987). Intrathoracic pressure is communicated through the vascular tree into the cranial cavity, leading to a transient increase in ICP and obstruction of venous outflow from the brain with, thus, increased venous pressure (Prabhakar et al., 2007). Reduced venous return to the heart causes ABP to decrease. Hence, cerebral perfusion pressure is reduced, leading to a reduction in cerebral blood flow (CBF). Cerebral autoregulation is a mechanism to maintain constant CBF. For this reason, cerebral autoregulation, after the decrease in CBF, immediately responds to reduce vascular resistance by dilating the cerebral arteries in order to facilitate blood flow and maintain stable CBF. At the same time, the heart rate is increased through the baroreflex (Eckberg, 1980; Looga, 1997), which restores normal ABP and accumulation of blood in the brain, since venous return is still diminished. Constant influx of blood with reduced outflow steadily increases ICP. With release of intrathoracic pressure, there is a significant drop of ABP (Stone et al., 1965), and ICP returns to normal. As a result, normal venous return is restored and more blood flows back into the heart, leading to a transient increase in cardiac output and overshoot in ABP. Since vascular resistance is still low, CBF overshoots as well.

**FIGURE 7:**
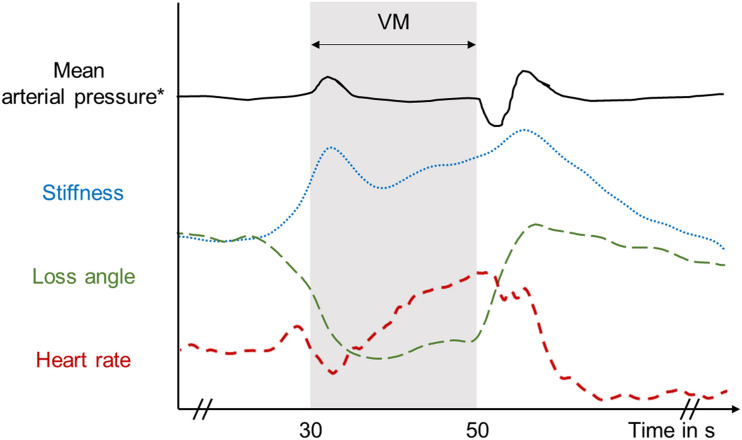
Diagram of average viscoelastic response with typical mean arterial pressure reported by Pstras et al. (2016) and heart rate variations during and after the Valsalva maneuver with inspiration before the maneuver as measured in this study.

The time curves of MRE parameters presented in Figure 7 suggest that stiffness (| G^∗^|) correlates with ICP while viscosity-related φ correlates with reduced venous outflow or cerebral perfusion pressure. Perfusion pressure is proportional to CBF normalized by mean vascular diameter (Hetzer et al., 2019) and, thus, decreases upon vasodilation with constant CBF. The ramp-up of | G^∗^| during the continuing VM phase seems to reflect the increasing heart rate and steady accumulation of blood in the brain, which drives ICP. By contrast, φ remains low throughout the VM phase as if viscous damping in brain tissue is lower when perfusion pressure is reduced. It is an intriguing result that possible ICP changes during VM can be indirectly monitored using rt-MMRE since non-invasive ICP measurement are still an unsolved problem. These findings could help to relate pathologically increased ICP to overall brain stiffness for clinical applications.

In previous work we observed an increase in φ of the brain due to hypercapnia (2% increase) (Hetzer et al., 2019) and arterial pulsation (0.5% increase) (Schrank et al., 2020b). In both studies, there was an increase in CBF with a concomitant increase in perfusion pressure while, as explained above, CBF in VM is, due to cerebral autoregulation, associated with a fairly constant CBF and reduced perfusion pressure. Together, the two rt-MMRE parameters, | G^∗^| and φ, provide complementary information on the concert of physical parameters involved in ICP autoregulation.

Overall, our baseline parameters of brain viscoelasticity are in good agreement with previously reported values acquired in similar frequency ranges (Hetzer et al., 2018, 2019; Schrank et al., 2020b). We observed no significant differences in the responses of rt-MMRE parameters to VM between the 31 and 42 Hz regimens. This consistency of multifrequency data further validates the technique of rt-MMRE. Furthermore, this observation indicates that the poroelastic response of brain tissue (Lilaj et al., 2020) is similar at 30 and 40 Hz (McGarry et al., 2015). Additional validation of rt-MMRE was obtained by reference experiments performed during breath-holds but without sustained VM. BH-in induced a similar increase in stiffness and decrease in φ as observed during VM. Thus, from an MRE perspective, deep inspiration followed by breath-holding induces effects similar to a *light* VM. Otherwise, no such changes were observed in BH-ex, rendering this maneuver neutral with regard to ICP. Nevertheless, even BH-ex had some small effect on MRE parameters, which, notably, were not correlated to changes in either SNR or WSNR. Also, analysis of CSF volume and ventricle size did not reveal any significant correlation with VM. Previous work by us and others showed that total brain volume increases due to VM by approximately 3% while ventricle volume shrinks by 20% (Ertl-Wagner et al., 2001; Mousavi et al., 2014). In contrast to these studies, our subjects were instructed to perform a moderate Valsalva maneuver to minimize variations in thoracic pressure, muscle strain, and head position.

In our previous work we used ultrasound time-harmonic elastography in a temporal bone window to acquire VM-induced rapid changes in shear wave speed in the temporal lobes of healthy volunteers (Tzschatzsch et al., 2018). Effect sizes in that region were higher (10.8 ± 2.5%) than revealed by MRE in the full brain tissue slice. It should be noted that the regions covered by our current study do not correspond to the medial temporal gyrus addressed by transtemporal time-harmonic elastography, which makes a direct comparison of effect sizes between the two studies difficult. To analyze the spatial representation of viscoelasticity changes we performed automatic image segmentation using MNI-based registration as well as voxel-wise correlation analysis based on a boxcar function. No significant patterns of viscoelasticity changes could be detected. A more detailed analysis of the spatiotemporal representation of brain viscoelasticity in response to the VM is warranted.

Our study has limitations. The nature of stroboscopic sampling of vibrations by steady-state single-shot acquisitions limited our technique to 2D wave field sampling including three encoding components. This intrinsic limitation of rt-MMRE can currently not be overcome by a multishot variant because VM is a non-periodic event and cannot be repeated with enough temporal reliability. Consequently, our multi-frequency inversion technique was entirely 2D, which may have led to variability due to different slice positioning and oblique intersection of 3D shear wavefields rendering our values as effective viscoelasticity parameters. Nevertheless, our conclusions are drawn from group values in two different frequency regimens. The fact that these values changed with statistical significance in VM, while neither SNR nor anatomy changed, emphasizes the robustness of the observed MRE effects. Furthermore, our data could be used for suppression of bulk waves based on the in-plane curl component. However, this curl-analysis did not provide more consistent values than our standard MDEV inversion with respect to confidence intervals and statistical power. Finally, 2D brain MRE has a long tradition in disease detection (Wuerfel et al., 2010; Streitberger et al., 2012, 2017, 2020; Lipp et al., 2013, 2018; Fehlner et al., 2016; Gerischer et al., 2018) as well as in the study of brain physiology (Sack et al., 2009, 2011; Schrank et al., 2020b; Herthum et al., 2021). It remains to be determined whether single-frequency 3D MRE can provide similarly consistent clinical and physiological brain data. Instead, as shown herein, unintentional breath-holds may affect 3D MRE due to long scan times. Generally, current MRE techniques cannot account for poroelasticity, heterogeneity, hyperelasticity, anisotropy and temporal variations of brain tissue at the same time. Therefore, to date *all* values measured by brain MRE should be considered as effective parameters.

In summary, we studied the viscoelastic response of the human brain to breathing and the Valsalva maneuver using a novel real-time multifrequency MRE technique. Significant increases in brain stiffness and decreases in φ due to VM were observed with use of two different frequency regimens. Control experiments showed that breath-holds after inhalation induce a response similar to VM but with a smaller effect size. By contrast, breath-holds after exhalation had the smallest effects on cerebral MRE parameters. The time courses we report here provide a reference for the VM response in healthy subjects and might be of value for studying dysfunctional autoregulation as associated with various neurological diseases. rt-MMRE is a fast technique which can provide consistent imaging markers of brain viscoelasticity within a fraction of a minute.

## Data Availability Statement

The original contributions presented in the study are included in the article/Supplementary Material, further inquiries can be directed to the corresponding author/s.

## Ethics Statement

The studies involving human participants were reviewed and approved by the Ethics committee of Charité – Universitätsmedizin Berlin in accordance with the Ethical Principles for Medical Research Involving Human Subjects of the World Medical Association Declaration of Helsinki. The patients/participants provided their written informed consent to participate in this study.

## Author Contributions

HH carried out all experiments and contributed to all parts of the manuscript. MS assisted in interpreting the results with regard to physiological changes and statistical analysis. FS, HT, and SH helped to carry out the data processing and verified the results. CW and JP carried out the MRI sequence implementation and image reconstruction. SG and HN contributed to the experimental setup. JB helped supervise the project and constructed the actuation system. IS designed and directed the project and aided in interpreting the results. All authors provided critical feedback and helped shape the research and manuscript.

## Conflict of Interest

JP was employed by company Siemens Healthcare GmbH, Erlangen, Germany. The remaining authors declare that the research was conducted in the absence of any commercial or financial relationships that could be construed as a potential conflict of interest.

## References

[B1] Arani A. Min H. K. Fattahi N. Wetjen N. M. Trzasko J. D. Manduca A. (2018). Acute pressure changes in the brain are correlated with MR elastography stiffness measurements: initial feasibility in an in vivo large animal model. *Magn. Reson. Med.* 79 1043–1051. 10.1002/mrm.26738 28488326 PMC5811891

[B2] Arani A. Murphy M. C. Glaser K. J. Manduca A. Lake D. S. Kruse S. A. (2015). Measuring the effects of aging and sex on regional brain stiffness with MR elastography in healthy older adults. *Neuroimage* 111 59–64. 25698157 10.1016/j.neuroimage.2015.02.016 PMC4387012

[B3] Barnhill E. Hollis L. Sack I. Braun J. Hoskins P. R. Pankaj P. (2017). Nonlinear multiscale regularisation in MR elastography: towards fine feature mapping. *Med. Image Anal.* 35 133–145. 10.1016/j.media.2016.05.012 27376240

[B4] Bertalan G. Boehm-Sturm P. Schreyer S. Morr A. S. Steiner B. Tzschatzsch H. (2019a). The influence of body temperature on tissue stiffness, blood perfusion, and water diffusion in the mouse brain. *Acta Biomater.* 96 412–420. 10.1016/j.actbio.2019.06.034 31247381

[B5] Bertalan G. Guo J. Tzschatzsch H. Klein C. Barnhill E. Sack I. (2019b). Fast tomoelastography of the mouse brain by multifrequency single-shot MR elastography. *Magn. Reson. Med.* 81 2676–2687. 10.1002/mrm.27586 30393887

[B6] Bertalan G. Klein C. Schreyer S. Steiner B. Kreft B. Tzschatzsch H. (2020). Biomechanical properties of the hypoxic and dying brain quantified by magnetic resonance elastography. *Acta Biomater.* 101 395–402. 10.1016/j.actbio.2019.11.011 31726251

[B7] Bilston L. E. (2002). The effect of perfusion on soft tissue mechanical properties: a computational model. *Comput. Methods Biomech. Biomed. Eng.* 5 283–290. 10.1080/10255840290032658 12186707

[B8] Chatelin S. Humbert-Claude M. Garteiser P. Ricobaraza A. Vilgrain V. Van Beers B. E. (2015). Cannabinoid receptor activation in the juvenile rat brain results in rapid biomechanical alterations: neurovascular mechanism as a putative confounding factor. *J. Cereb. Blood Flow Metab.* 36:954–964. 10.1177/0271678x15606923 26661178 PMC4853836

[B9] Donoho D. L. Johnstone I. M. Kerkyacharian G. Picard D. (1995). Wavelet shrinkage: asymptopia? *J. R. Statist. Soc. Ser. B (Methodological)* 57 301–337. 10.1111/j.2517-6161.1995.tb02032.x

[B10] Eckberg D. L. (1980). Parasympathetic cardiovascular control in human disease: a critical review of methods and results. *Am. J. Physiol.* 239 H581–H593. 7001926 10.1152/ajpheart.1980.239.5.H581

[B11] Elisberg E. I. (1963). Heart rate response to the valsalva maneuver as a test of circulatory integrity. *JAMA* 186 200–205. 10.1001/jama.1963.03710030040006 14057108

[B12] Ertl-Wagner B. B. Lienemann A. Reith W. Reiser M. F. (2001). Demonstration of periventricular brain motion during a Valsalva maneuver: description of technique, evaluation in healthy volunteers and first results in hydrocephalic patients. *Eur. Radiol.* 11 1998–2003. 11702134 10.1007/s003300100941

[B13] Fehlner A. Behrens J. R. Streitberger K. J. Papazoglou S. Braun J. Bellmann-Strobl J. (2016). Higher-resolution MR elastography reveals early mechanical signatures of neuroinflammation in patients with clinically isolated syndrome. *J. Magn. Reson. Imag.* 44 51–58. 10.1002/jmri.25129 26714969

[B14] Gerischer L. M. Fehlner A. Kobe T. Prehn K. Antonenko D. Grittner U. (2018). Combining viscoelasticity, diffusivity and volume of the hippocampus for the diagnosis of Alzheimer’s disease based on magnetic resonance imaging. *Neuroimage Clin.* 18 485–493. 29527504 10.1016/j.nicl.2017.12.023 PMC5842309

[B15] Giulioni M. Ursino M. Alvisi C. (1988). Correlations among intracranial pulsatility, intracranial hemodynamics, and transcranial doppler wave form: literature review and hypothesis for future studies. *Neurosurgery* 22 807–812. 10.1227/00006123-198805000-00001 3288898

[B16] Greitz D. Wirestam R. Franck A. Nordell B. Thomsen C. Stahlberg F. (1992). Pulsatile brain movement and associated hydrodynamics studied by magnetic resonance phase imaging. the monro-Kellie doctrine revisited. *Neuroradiology* 34 370–380. 10.1007/bf00596493 1407513

[B17] Guo J. Bertalan G. Meierhofer D. Klein C. Schreyer S. Steiner B. (2019). Brain maturation is associated with increasing tissue stiffness and decreasing tissue fluidity. *Acta Biomater.* 99 433–442. 10.1016/j.actbio.2019.08.036 31449927

[B18] Guyton A. Hall J. (2006). *Textbook of Medical Physiology*, 11th ed. Elsevier Inc., Amsterdam.

[B19] Hatt A. Cheng S. Tan K. Sinkus R. Bilston L. E. (2015). MR elastography can be used to measure brain stiffness changes as a result of altered cranial venous drainage during jugular compression. *AJNR Am. J. Neuroradiol.* 36 1971–1977. 10.3174/ajnr.a4361 26045579 PMC7965045

[B20] Herthum H. Dempsey S. C. H. Samani A. Schrank F. Shahryari M. Warmuth C. (2021). Superviscous properties of the in vivo brain at large scales. *Acta Biomater.* 121 393–404. 10.1016/j.actbio.2020.12.027 33326885

[B21] Hetzer S. Birr P. Fehlner A. Hirsch S. Dittmann F. Barnhill E. (2018). Perfusion alters stiffness of deep gray matter. *J. Cereb. Blood Flow Metab.* 38 116–125. 28151092 10.1177/0271678X17691530 PMC5757437

[B22] Hetzer S. Dittmann F. Bormann K. Hirsch S. Lipp A. Wang D. J. (2019). Hypercapnia increases brain viscoelasticity. *J. Cereb. Blood Flow Metab.* 39 2445–2455. 30182788 10.1177/0271678X18799241 PMC6893988

[B23] Hirsch S. Braun J. Sack I. (2017). *Magnetic Resonance Elastography: Physical Background and Medical Applications.* Wiley-VCH: Weinheim.

[B24] Hirsch S. Guo J. Reiter R. Papazoglou S. Kroencke T. Braun J. (2014). MR elastography of the liver and the spleen using a piezoelectric driver, single-shot wave-field acquisition, and multifrequency dual parameter reconstruction. *Magn. Reson. Med.* 71 267–277. 10.1002/mrm.24674 23413115

[B25] Hiscox L. V. Johnson C. L. Barnhill E. McGarry M. D. J. Huston J. van Beek E. J. R. (2016). Magnetic resonance elastography (MRE) of the human brain: technique, findings and clinical applications. *Phys. Med. Biol.* 61 R401–R437. 27845941 10.1088/0031-9155/61/24/R401

[B26] Ipek-Ugay S. Tzschaetzsch H. Fischer T. Braun J. Sack I. (2017). Physiological reduction of hepatic venous blood flow by *Valsalva maneuver* decreases liver stiffness. *J. Ultrasound Med.* 36 1305–1311. 28319252 10.7863/ultra.16.07046

[B27] Jamin Y. Boult J. K. R. Li J. Popov S. Garteiser P. Ulloa J. L. (2015). Exploring the biomechanical properties of brain malignancies and their pathologic determinants in vivo with magnetic resonance elastography. *Cancer Res.* 75 1216–1224. 10.1158/0008-5472.can-14-1997 25672978 PMC4384983

[B28] Juge L. Pong A. C. Bongers A. Sinkus R. Bilston L. E. Cheng S. (2016). Changes in rat brain tissue microstructure and stiffness during the development of experimental obstructive hydrocephalus. *PLoS One* 11:e0148652. 10.1371/journal.pone.0148652 26848844 PMC4743852

[B29] Klein C. Hain E. G. Braun J. Riek K. Mueller S. Steiner B. (2014). Enhanced adult neurogenesis increases brain stiffness: in vivo magnetic resonance elastography in a mouse model of dopamine depletion. *PLoS One* 9:e92582. 10.1371/journal.pone.0092582 24667730 PMC3965445

[B30] Kreft B. Tzschatzsch H. Schrank F. Bergs J. Streitberger K. J. Waldchen S. (2020). Time-Resolved response of cerebral stiffness to hypercapnia in humans. *Ultrasound Med. Biol.* 46 936–943. 10.1016/j.ultrasmedbio.2019.12.019 32001088

[B31] Lan P. S. Glaser K. J. Ehman R. L. Glover G. H. (2020). Imaging brain function with simultaneous BOLD and viscoelasticity contrast: fMRI/fMRE. *Neuroimage* 211:116592. 10.1016/j.neuroimage.2020.116592 32014553 PMC7153752

[B32] Lilaj L. Fischer T. Guo J. Braun J. Sack I. Hirsch S. (2020). Separation of fluid and solid shear wave fields and quantification of coupling density by magnetic resonance poroelastography. *Magn. Reson. Med.* 85 1655–1668. 10.1002/mrm.28507 32902011

[B33] Linninger A. A. Tsakiris C. Zhu D. C. Xenos M. Roycewicz P. Danziger Z. (2005). Pulsatile cerebrospinal fluid dynamics in the human brain. *IEEE Trans. Biomed. Eng.* 52 557–565. 10.1109/tbme.2005.844021 15825857

[B34] Lipp A. Skowronek C. Fehlner A. Streitberger K. J. Braun J. Sack I. (2018). Progressive supranuclear palsy and idiopathic Parkinson’s disease are associated with local reduction of in vivo brain viscoelasticity. *Eur. Radiol.* 28 3347–3354. 10.1007/s00330-017-5269-y 29460073

[B35] Lipp A. Trbojevic R. Paul F. Fehlner A. Hirsch S. Scheel M. (2013). Cerebral magnetic resonance elastography in supranuclear palsy and idiopathic Parkinson’s disease. *Neuroimage Clin.* 3 381–387. 10.1016/j.nicl.2013.09.006 24273721 PMC3814959

[B36] Looga R. (1997). Reflex cardiovascular responses to lung inflation: a review. *Respir Physiol.* 109 95–106. 10.1016/s0034-5687(97)00049-2 9299641

[B37] Lustig M. Pauly J. M. (2010). SPIRiT: Iterative self-consistent parallel imaging reconstruction from arbitrary k-space. *Magn. Reson. Med.* 64 457–471. 10.1002/mrm.22428 20665790 PMC2925465

[B38] McGarry M. D. Johnson C. L. Sutton B. P. Georgiadis J. G. Van Houten E. E. Pattison A. J. (2015). Suitability of poroelastic and viscoelastic mechanical models for high and low frequency MR elastography. *Med. Phys.* 42 947–957. 10.1118/1.4905048 25652507 PMC4312344

[B39] Meyer T. Tzschätzsch H. Braun J. Kalra P. Kolipaka A. Sack I. (2019). “Online platform for extendable server-based processing of magnetic resonance elastography data,” in *Proceedings of the 27st Annual Meeting of ISMRM* (Montreal, QC, Canada), 3966.

[B40] Mousavi S. R. Fehlner A. Streitberger K. J. Braun J. Samani A. Sack I. (2014). Measurement of in vivo cerebral volumetric strain induced by the *Valsalva maneuver*. *J. Biomech.* 47 1652–1657. 10.1016/j.jbiomech.2014.02.038 24656483

[B41] Munder T. Pfeffer A. Schreyer S. Guo J. Braun J. Sack I. (2018). MR elastography detection of early viscoelastic response of the murine hippocampus to amyloid β accumulation and neuronal cell loss due to Alzheimer’s disease. *J. Magn. Reson. Imag.* 47 105–114. 10.1002/jmri.25741 28422391

[B42] Murphy M. C. Cogswell P. M. Trzasko J. D. Manduca A. Senjem M. L. Meyer F. B. (2020). Identification of normal pressure hydrocephalus by disease-specific patterns of brain stiffness and damping ratio. *Invest. Radiol.* 55 200–208. 10.1097/rli.0000000000000630 32058331 PMC7681913

[B43] Murphy M. C. Curran G. L. Glaser K. J. Rossman P. J. Huston J. 3rd Poduslo J. F. (2012). Magnetic resonance elastography of the brain in a mouse model of Alzheimer’s disease: initial results. *Magn. Reson. Imag.* 30 535–539. 10.1016/j.mri.2011.12.019 22326238 PMC3433281

[B44] Murphy M. C. Huston J. IIIrd. Jack C. R. Jr. Glaser K. J. Manduca A. (2011). Decreased brain stiffness in Alzheimer’s disease determined by magnetic resonance elastography. *J. Magn. Reson. Imag.* 34 494–498. 10.1002/jmri.22707 21751286 PMC3217096

[B45] Murphy M. C. Jones D. T. Jack C. R. Jr. Glaser K. J. Senjem M. L. (2016). Regional brain stiffness changes across the Alzheimer’s disease spectrum. *Neuroimage Clin.* 10 283–290. 10.1016/j.nicl.2015.12.007 26900568 PMC4724025

[B46] Papazoglou S. Hirsch S. Braun J. Sack I. (2012). Multifrequency inversion in magnetic resonance elastography. *Phys. Med. Biol.* 57 2329–2346. 10.1088/0031-9155/57/8/2329 22460134

[B47] Parker K. J. (2014). A microchannel flow model for soft tissue elasticity. *Phys. Med. Biol.* 59 4443–4457. 10.1088/0031-9155/59/15/4443 25049224

[B48] Parker K. J. (2017). Are rapid changes in brain elasticity possible? *Phys. Med. Biol.* 62 7425–7439. 10.1088/1361-6560/aa8380 28766505

[B49] Patz S. Fovargue D. Schregel K. Nazari N. Palotai M. Barbone P. E. (2019). Imaging localized neuronal activity at fast time scales through biomechanics. *Sci. Adv.* 5:eaav3816. 10.1126/sciadv.aav3816 31001585 PMC6469937

[B50] Penny W. D. Friston K. J. Ashburner J. T. Kiebel S. J. Nichols T. E. (2011). *Statistical Parametric Mapping: the Analysis of Functional Brain Images.* Elsevier: Amsterdam.

[B51] Perrinez P. R. Kennedy F. E. Van Houten E. E. Weaver J. B. Paulsen K. D. (2009). Modeling of soft poroelastic tissue in time-harmonic MR elastography. *IEEE Trans. Biomed. Eng.* 56 598–608. 10.1109/tbme.2008.2009928 19272864 PMC2857336

[B52] Prabhakar H. Bithal P. K. Suri A. Rath G. P. Dash H. H. (2007). Intracranial pressure changes during *Valsalva manoeuvre* in patients undergoing a neuroendoscopic procedure. *Minim. Invasive Neurosurg.* 50 98–101. 10.1055/s-2007-982505 17674296

[B53] Pstras L. Thomaseth K. Waniewski J. Balzani I. Bellavere F. (2016). The Valsalva manoeuvre: physiology and clinical examples. *Acta Physiol. (Oxf)* 217 103–119. 10.1111/apha.12639 26662857

[B54] Reiss-Zimmermann M. Streitberger K. J. Sack I. Braun J. Arlt F. Fritzsch D. (2015). High resolution imaging of viscoelastic properties of intracranial tumours by multi-frequency magnetic resonance elastography. *Clin. Neuroradiol.* 25 371–378. 10.1007/s00062-014-0311-9 24916129

[B55] Riek K. Millward J. M. Hamann I. Mueller S. Pfueller C. F. Paul F. (2012). Magnetic resonance elastography reveals altered brain viscoelasticity in experimental autoimmune encephalomyelitis. *Neuroimage Clin.* 1 81–90. 10.1016/j.nicl.2012.09.003 24179740 PMC3757734

[B56] Rump J. Klatt D. Braun J. Warmuth C. Sack I. (2007). Fractional encoding of harmonic motions in MR elastography. *Magn. Reson. Med.* 57 388–395. 10.1002/mrm.21152 17260354

[B57] Sack I. Beierbach B. Wuerfel J. Klatt D. Hamhaber U. Papazoglou S. (2009). The impact of aging and gender on brain viscoelasticity. *Neuroimage* 46 652–657. 10.1016/j.neuroimage.2009.02.040 19281851

[B58] Sack I. Streitberger K. J. Krefting D. Paul F. Braun J. (2011). The influence of physiological aging and atrophy on brain viscoelastic properties in humans. *PLos One* 6:e23451. 10.1371/journal.pone.0023451 21931599 PMC3171401

[B59] Schmid Daners M. Knobloch V. Soellinger M. Boesiger P. Seifert B. Guzzella L. (2012). Age-specific characteristics and coupling of cerebral arterial inflow and cerebrospinal fluid dynamics. *PLoS One* 7:e37502. 10.1371/journal.pone.0037502 22666360 PMC3364266

[B60] Schrank F. Warmuth C. Gorner S. Meyer T. Tzschatzsch H. Guo J. (2020a). Real-time MR elastography for viscoelasticity quantification in skeletal muscle during dynamic exercises. *Magn. Reson. Med.* 84 103–114. 10.1002/mrm.28095 31774210

[B61] Schrank F. Warmuth C. Tzschatzsch H. Kreft B. Hirsch S. Braun J. (2020b). Cardiac-gated steady-state multifrequency magnetic resonance elastography of the brain: effect of cerebral arterial pulsation on brain viscoelasticity. *J. Cereb. Blood Flow Metab.* 40 991–1001. 10.1177/0271678x19850936 31142226 PMC7181097

[B62] Schregel K. Nazari N. Nowicki M. O. Palotai M. Lawler S. E. Sinkus R. (2018). Characterization of glioblastoma in an orthotopic mouse model with magnetic resonance elastography. *NMR Biomed.* 31:e3840. 10.1002/nbm.3840 29193449 PMC6538416

[B63] Schregel K. Wuerfel E. Garteiser P. Gemeinhardt I. Prozorovski T. Aktas O. (2012). Demyelination reduces brain parenchymal stiffness quantified in vivo by magnetic resonance elastography. *Proc. Natl. Acad. Sci. U.S.A.* 109 6650–6655. 10.1073/pnas.1200151109 22492966 PMC3340071

[B64] Selesnick I. W. Baraniuk R. G. Kingsbury N. G. (2005). The dual-tree complex wavelet transform. *IEEE Signal Process. Magazine* 22 123–151.

[B65] Shahryari M. Meyer T. Warmuth C. Herthum H. Bertalan G. Tzschätzsch H. (2020). Reduction of breathing artifacts in multifrequency magnetic resonance elastography of the abdomen. *Magn. Reson. Med.* 85:1962–1973. 10.1002/mrm.28558 33104294

[B66] Simon M. Guo J. Papazoglou S. Scholand-Engler H. Erdmann C. Melchert U. (2013). Non-invasive characterization of intracranial tumors by MR-Elastography. *N. J. Phys.* 15:085024. 10.1088/1367-2630/15/8/085024

[B67] Smith S. A. Salih M. M. Littler W. A. (1987). Assessment of beat to beat changes in cardiac output during the *Valsalva manoeuvre* using electrical bioimpedance cardiography. *Clin. Sci. (Lond)* 72 423–428. 10.1042/cs0720423 3829590

[B68] Stone D. J. Lyon A. F. Teirstein A. S. (1965). A reappraisal of the circulatory effects of the *Valsalva maneuver*. *Am. J. Med.* 39 923–933. 10.1016/0002-9343(65)90114-2 5853043

[B69] Streitberger K. J. Fehlner A. Pache F. Lacheta A. Papazoglou S. Bellmann-Strobl J. (2017). Multifrequency magnetic resonance elastography of the brain reveals tissue degeneration in neuromyelitis optica spectrum disorder. *Eur. Radiol.* 27 2206–2215. 10.1007/s00330-016-4561-6 27572811

[B70] Streitberger K. J. Lilaj L. Schrank F. Braun J. Hoffmann K. T. Reiss-Zimmermann M. (2020). How tissue fluidity influences brain tumor progression. *Proc. Natl. Acad. Sci. U.S.A.* 117 128–134. 10.1073/pnas.1913511116 31843897 PMC6955323

[B71] Streitberger K. J. Reiss-Zimmermann M. Freimann F. B. Bayerl S. Guo J. Arlt F. (2014). High-resolution mechanical imaging of glioblastoma by multifrequency magnetic resonance elastography. *PLoS One* 9:e110588. 10.1371/journal.pone.0110588 25338072 PMC4206430

[B72] Streitberger K. J. Sack I. Krefting D. Pfuller C. Braun J. Paul F. (2012). Brain viscoelasticity alteration in chronic-progressive multiple sclerosis. *PLoS One* 7:e29888. 10.1371/journal.pone.0029888 22276134 PMC3262797

[B73] Streitberger K. J. Wiener E. Hoffmann J. Freimann F. B. Klatt D. Braun J. (2011). In vivo viscoelastic properties of the brain in normal pressure hydrocephalus. *NMR Biomed.* 24 385–392. 20931563 10.1002/nbm.1602

[B74] Tully B. Ventikos Y. (2011). Cerebral water transport using multiple-network poroelastic theory: application to normal pressure hydrocephalus. *J. Fluid Mechan.* 667 188–215. 10.1017/s0022112010004428

[B75] Tzschatzsch H. Kreft B. Schrank F. Bergs J. Braun J. Sack I. (2018). In vivo time-harmonic ultrasound elastography of the human brain detects acute cerebral stiffness changes induced by intracranial pressure variations. *Sci. Rep.* 8:17888. 10.1038/s41598-018-36191-9 PMC6297160 30559367

[B76] Wagshul M. E. Chen J. J. Egnor M. R. McCormack E. J. Roche P. E. (2006). Amplitude and phase of cerebrospinal fluid pulsations: experimental studies and review of the literature. *J. Neurosurg.* 104 810–819. 10.3171/jns.2006.104.5.810 16703889

[B77] Wagshul M. E. Eide P. K. Madsen J. R. (2011). The pulsating brain: a review of experimental and clinical studies of intracranial pulsatility. *Fluids Barriers CNS* 8:5. 10.1186/2045-8118-8-5 PMC3042979 21349153

[B78] Wang J. Shan Q. Liu Y. Yang H. Kuang S. He B. (2019). 3D MR elastography of hepatocellular carcinomas as a potential biomarker for predicting tumor recurrence. *J. Magn. Reson. Imag.* 49 719–730. 10.1002/jmri.26250 30260529 PMC6731763

[B79] Weickenmeier J. Kurt M. Ozkaya E. de Rooij R. Ovaert T. C. Ehman R. L. (2018). Brain stiffens post mortem. *J. Mech. Behav. Biomed. Mater.* 84 88–98. 10.1016/j.jmbbm.2018.04.009 29754046 PMC6751406

[B80] Wuerfel J. Paul F. Beierbach B. Hamhaber U. Klatt D. Papazoglou S. (2010). MR-elastography reveals degradation of tissue integrity in multiple sclerosis. *Neuroimage* 49 2520–2525. 10.1016/j.neuroimage.2009.06.018 19539039

[B81] Yin Z. Romano A. J. Manduca A. Ehman R. L. Huston J. 3rd. (2018). Stiffness and beyond: what MR elastography can tell us about brain structure and function under physiologic and pathologic conditions. *Top Magn. Reson. Imag.* 27 305–318. 10.1097/rmr.0000000000000178 30289827 PMC6176744

