# Supplementary material for: Real-Time Multifrequency MR Elastography of the Human Brain Reveals Rapid Changes in Viscoelasticity in Response to the Valsalva Maneuver
Source: Front Bioeng Biotechnol. 2021 May 5;9:666456. doi: 10.3389/fbioe.2021.666456 (PMC8131519; doi:10.3389/fbioe.2021.666456)
Supplement: Supplementary file 1 [file Data_Sheet_1.docx]

**Supplementary material to:**

**Real-time multifrequency MR elastography of the human brain reveals rapid changes in viscoelasticity in response to the Valsalva maneuver**

Helge Herthum^1^, Mehrgan Shahryari^2^, Heiko Tzschätzsch^2^, Felix Schrank^2^, Carsten Warmuth^2^, Steffen Görner^2^, Stefan Hetzer^3^, Hennes Neubauer^2^, Josef Pfeuffer^4^, Jürgen Braun^1^, Ingolf Sack^2,*^

^1^Institute of Medical Informatics, Charité – Universitätsmedizin Berlin, corporate member of Freie Universität Berlin, Humboldt-Universität zu Berlin, and Berlin Institute of Health, Berlin, Germany

^2^Department of Radiology, Charité – Universitätsmedizin Berlin, corporate member of Freie Universität Berlin, Humboldt-Universität zu Berlin, and Berlin Institute of Health, Berlin, Germany

^3^Berlin Center for Advanced Neuroimaging (BCAN), Berlin, Germany

^4^Application Development, Siemens Healthcare GmbH, Erlangen, Germany.

***Corresponding author:**

Ingolf Sack, PhD
Department of Radiology
Charité – Universitätsmedizin Berlin
Charitéplatz 1
10117 Berlin, Germany
Tel +49 30 450 539058
Ingolf.sack@charite.de

We present here the results of the Valsalva maneuver (VM) experiment at a second narrowband frequency regimen comprising 40.77, 41.67, 42.55 Hz (hereinafter, referred to as 42-Hz regimen) as a complementary evaluation of our findings. We did so in order to validate the overall consistency of MRE during the VM and to check if there is a noticeable influence of frequency. Despite different absolute values, the repetition of the VM experiment gave similar results compared to those described in the main manuscript at 31-Hz regimen. Moreover we present the group mean heart rate (HR) and additional descriptive statistics for the VM, breath-hold in inspiration (BH-in) and breath-hold at expiration (BH-ex).

Supplementary figure 1 shows group mean HR for each experiment. Qualitatively the course of the HR for the VM experiment at both frequency regimens was the same (see subfigure A and D). HR increased with deep inspiration (25 s) and dropped with onset of the VM (30 s). Next, HR starts to rise (30 – 50 s). With the end of the maneuver HR returned to normal or even below pre-exercise baseline values. BH-in showed a slight increase in HR similar to the VM (25 s) with deep inspiration. HR normalized throughout the breath-hold phase and is slightly lowered after the exercise (subfigure B). In the BH-ex experiment the HR did not show any changes with onset of the exercise but is slightly lowered with release and return to normal breathing as it can be observed in all experiments.


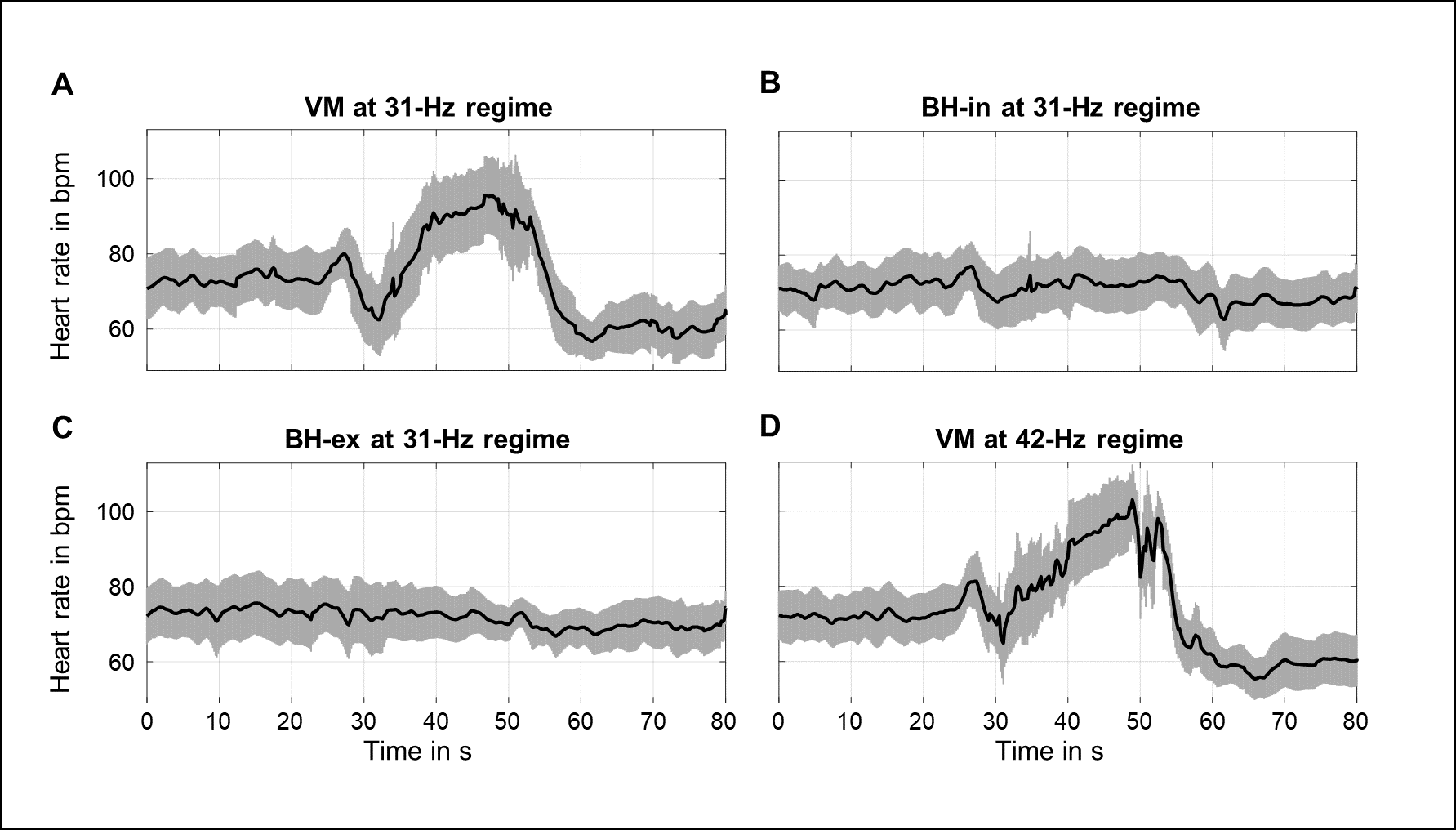


**Supplementary figure 1:** Group mean heart rate in bpm for each experiment. **A)** VM using the 31-Hz regimen (30.03, 30.91, 31.8 Hz). **B)** BH-in using the 31-Hz regimen. **C)** BH-ex using the 31-Hz regimen. **D)** VM using the 42-Hz regimen (40.77, 41.67, 42.55 Hz).

Supplementary figure 2 shows the spectral power of aliased driving frequencies of the 42-Hz regimen in addition to the 31-Hz regimen with wave images of three Cartesian field component and three vibration frequencies below. The higher frequencies contained less spectral power and with smaller wave amplitudes.


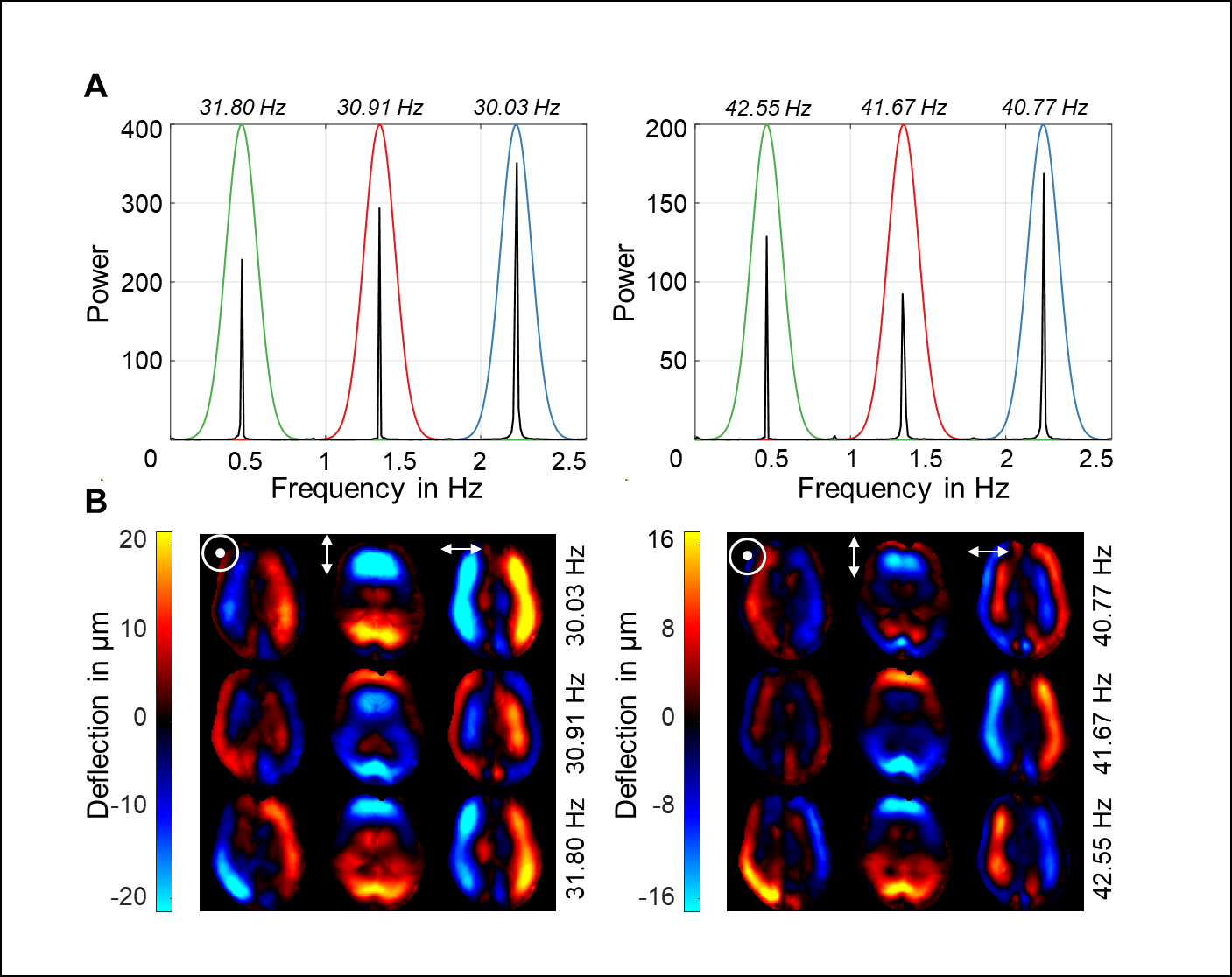


**Supplementary figure 2:** Representative Fourier power spectra with three aliased excitation frequencies for one motion-encoding component above and wave deflections for three encoding components and three vibration frequencies below. **A)** Power spectra for vibrations using the 31-Hz (left) and 42-Hz regimen (right). Color coding indicates the respective vibration frequency with Gaussian bandpass filter used for Hilbert transformation. The frequency axis is scaled from 0 to the Nyquist frequency in Hz, which is determined by the sampling rate of 5.4 Hz. Stroboscopic sampling of multiharmonic vibrations causes all frequencies to be aliased within this limited frequency window. **B)** Representative wave images after frequency decomposition for the three encoding components using the 31-Hz (left) and the 42-Hz regimens (right). (🖸,, denote deflections through-plane [head-to-feet], left-right, and up-down [anterior-posterior], respectively).

Supplementary figure 3 shows representative |G*| and φ maps in the 42-Hz regimen in addition to the 31-Hz regimen during the four phases of the experiment. Average |G*| was higher at 42-Hz than at 31-Hz as expected from the viscoelastic dispersion while φ was lower at higher frequencies.


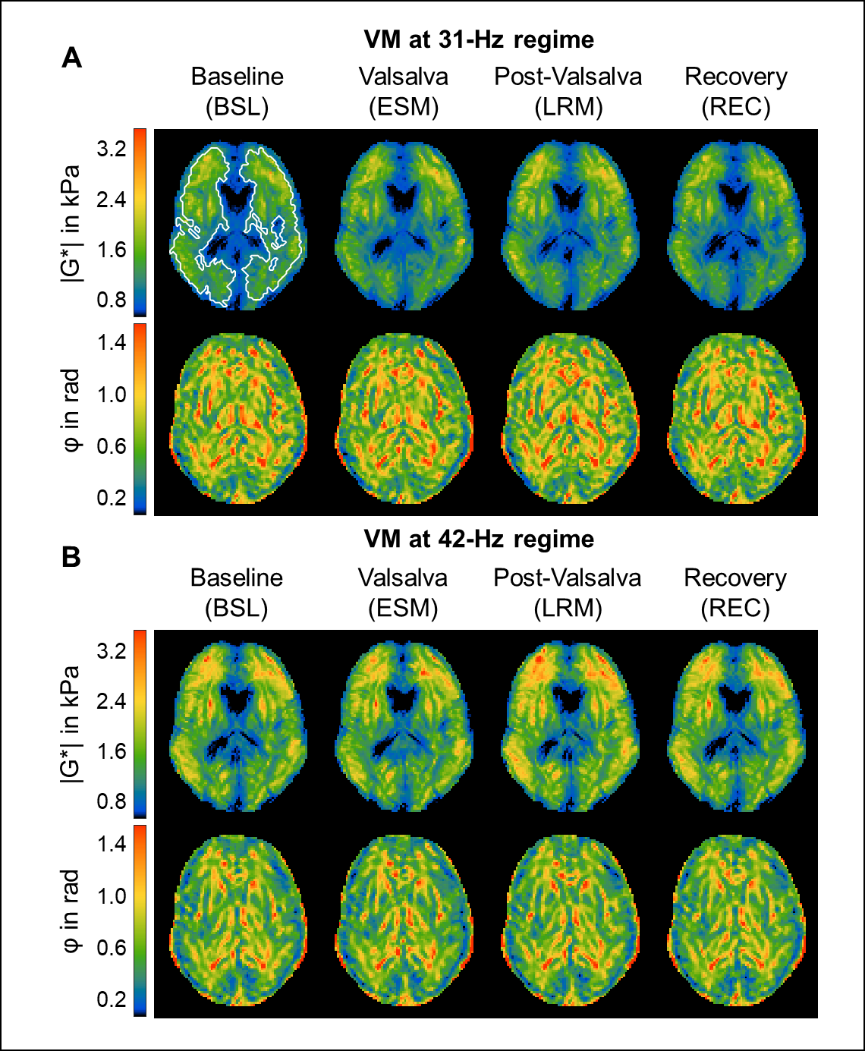


**Supplementary figure 3:** Representative rt-MMRE |G*| and φ maps of the in vivo human brain. **A)** Time-averaged |G*| and φ maps of one volunteer over the four phases (baseline (BSL), established maneuver (ESM), late response maneuver (LRM) and recovery (REC)) of the VM experiment using the 31-Hz regimen (30.03, 30.91, 31.8 Hz). **B)** Averaged |G*| and φ maps at 42-Hz regimen (40.77, 41.67, 42.55 Hz). Similar stiffening during the Valsalva maneuver (increases in most |G*| values) as in (A) is visible. The region of interest (ROI) is indicated by white lines. The same ROI was used for all phases, for the φ maps and for the 42-Hz regimen as well.

Averaged time courses of ∆|G*| and ∆φ at 42-Hz regimen in comparison the 31-Hz regimen are presented in supplementary Figure 4. As reported before, peak viscoelastic responses did not differ significantly in the two narrowband regimens. In accordance, qualitative time courses match well.

For 42-Hz regimen, group mean VM values showed an early overshoot of |G*| 2.4 ± 1.2 s after the onset of the maneuver with peak values of 4.4 ± 3.8% (in average: 61 ± 63 Pa, P < 0.001). A second overshoot of |G*| occurred 5.5 ± 2.0 s after the end of the VM with peak values of 7.2 ± 3.4% (in average: 124 ± 72 Pa, P < 0.01) above baseline. φ was reduced by -2.2 ± 2.1% (in average: -0.015 ± 0.014 rad, P < 0.01) during the entire VM without noticeable peak values.


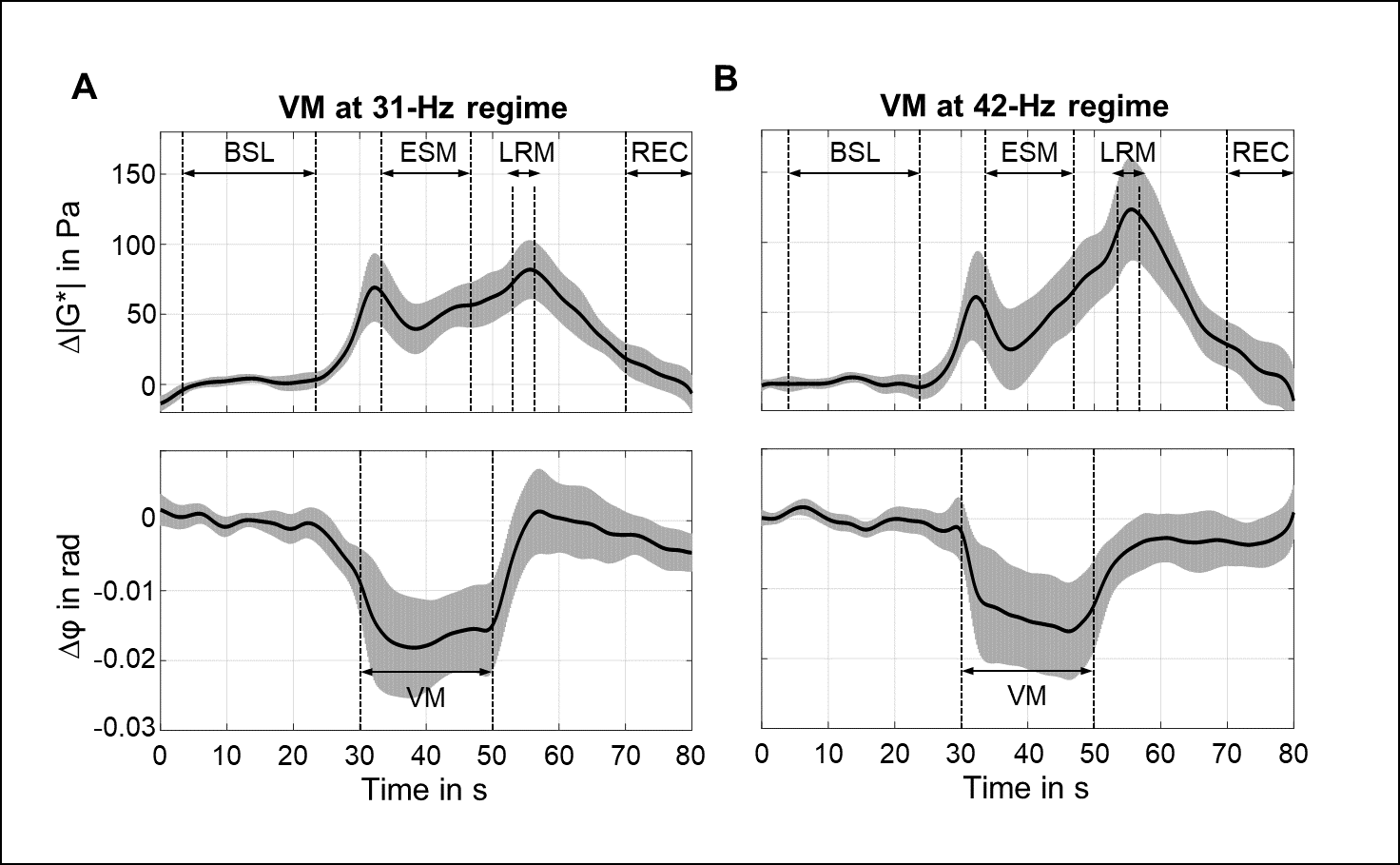


**Supplementary figure 4:** Time courses of group mean values of ∆|G*| (top of subfigures) and ∆φ values (bottom of subfigures). The gray areas show 95% confidence intervals. For Valsalva maneuver (VM), timing was as follows: breath-hold in inspiration (BH-in) at 25 s, start of VM at 30 s, stop of VM at 50 s. **A)** VM using the 31-Hz regimen (30.03, 30.91, 31.8 Hz). **B)** VM using the 42-Hz regimen (40.77, 41.67, 42.55 Hz).

Supplementary figure shows boxplots with median effects for different states of the maneuver for |G*| and φ at 42-Hz regimen in addition to 31-Hz regimen. The significance levels, indicated by asterisks, were determined from a linear mixed model analysis with varying intercept and participants as random effect. In the VM, averaged |G*| values changed in all phases of the experiment (range: 1953 to 2072 Pa) with significance levels indicated in the figure. Averaged φ values changed in both cases from BSL to ESM and again from ESM to LRM (range: 0.674 to 0.686 rad). Descriptive statistics for the repetition of the VM experiment in the 42-Hz regimen and BH-experiments are given in Supplementary table 2a-c. A negative correlation between BSL-|G*| values and age at 42-Hz regimen (0.8% per year, R = -0.64, P < 0.005) was observed.


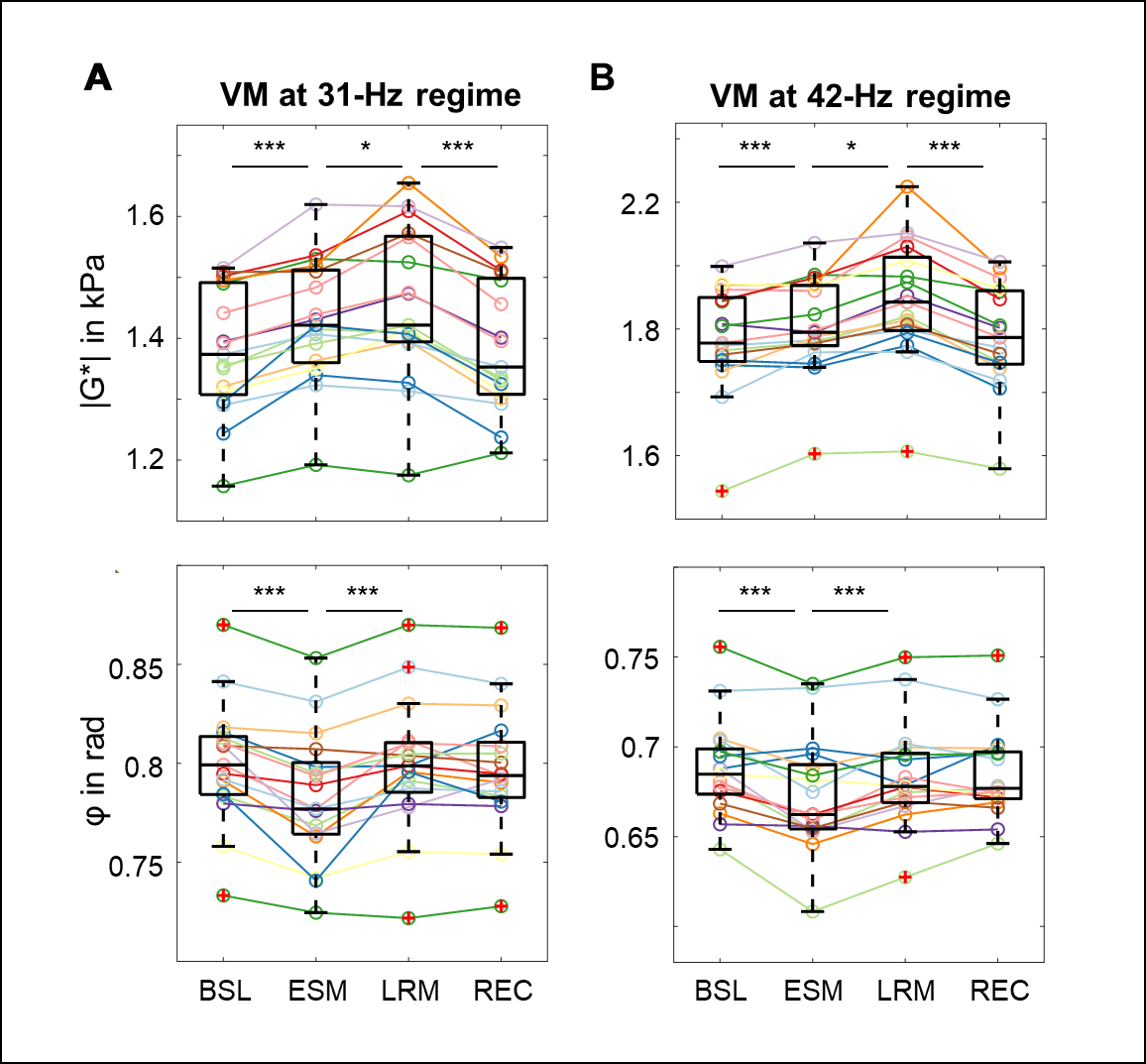


**Supplementary figure 5:** Group values as boxplots for the absolute values of |G*| (top) and φ (bottom) in each phase illustrate the changes in viscoelastic properties induced by VM during the two frequency regimens for each phase (baseline (BSL), established maneuver (ESM), late response maneuver (LRM), recovery (REC)). **A)** Valsalva maneuver (VM) using the 31-Hz regimen. **B)** VM using the 42-Hz regimen. Asterisks at the top demarcate significant changes in |G*| and φ which were determined from a linear mixed-effects model with varying intercept. |G*| and φ assigned dependent variables and the individual phases as independent variables. Participants were assigned as random effect, and P values were calculated using Tukey’s post hoc test with Bonferroni correction for multiple comparisons. (*P < .05, ***P < .001)

Supplementary table 1a-c present mean |G*| and φ values for the individual phases and each participant for the experiments: VM in the 42-Hz regimen (1a), BH-in (1b) and BH-ex (1c) similar to Table 2 in the main manuscript.

|  | Mean \|G*\| (SD) in Pa | | | | Mean φ (SD) in rad | | | |
| --- | --- | --- | --- | --- | --- | --- | --- | --- |
| **ID** | BSL | ESM | LRM | REC | BSL | ESM | LRM | REC |
| **1** | 1947  (7) | 1965  (19) | 1997  (8) | 1942  (16) | 0.731 (0.002) | 0.733 (0.005) | 0.738 (0.002) | 0.727 (0.002) |
| **2** | 1887  (17) | 1879  (8) | 1948  (14) | 1812  (18) | 0.688 (0.002) | 0.696 (0.009) | 0.679 (0.001) | 0.701 (0.005) |
| **3** | 1933  (15) | 1957  (16) | 2040  (8) | 1895  (10) | 0.681 (0.001) | 0.653 (0.003) | 0.675 (0.007) | 0.677 (0.003) |
| **4** | 2086  (12) | 2171  (36) | 2166  (5) | 2118  (12) | 0.756 (0.004) | 0.735 (0.003) | 0.75 (0.002) | 0.751 (0.002) |
| **5** | 2125  (5) | 2120  (43) | 2293  (35) | 2161  (11) | 0.68 (0.002) | 0.659 (0.002) | 0.683 (0.002) | 0.674 (0.001) |
| **6** | 2091  (17) | 2161  (23) | 2260  (18) | 2094  (17) | 0.675 (0.003) | 0.662 (0.001) | 0.678 (0.004) | 0.672 (0.002) |
| **7** | 1866  (7) | 1975  (16) | 2027  (13) | 1876  (17) | 0.705 (0.001) | 0.688 (0.008) | 0.7 (0.006) | 0.699 (0.003) |
| **8** | 2139  (4) | 2140  (37) | 2449  (17) | 2192  (10) | 0.663 (0.002) | 0.646 (0.001) | 0.662 (0.001) | 0.669 (0.002) |
| **9** | 2198  (7) | 2272  (15) | 2303  (10) | 2212  (15) | 0.688 (0.004) | 0.653 (0.008) | 0.667 (0.004) | 0.678 (0.004) |
| **10** | 2016  (6) | 1990  (12) | 2105  (16) | 2003  (18) | 0.657 (0.002) | 0.656 (0.003) | 0.653 (0.001) | 0.654 (0.002) |
| **11** | 1819  (8) | 1793  (26) | 1928  (14) | 1829  (24) | 0.693 (0.002) | 0.69 (0.002) | 0.687 (0.003) | 0.689 (0.002) |
| **12** | 1855  (15) | 1817  (25) | 1943  (17) | 1859  (14) | 0.654 (0.003) | 0.67 (0.01) | 0.659 (0.001) | 0.646 (0.003) |
| **13** | 2142  (6) | 2136  (22) | 2216  (7) | 2128  (12) | 0.685 (0.002) | 0.682 (0.007) | 0.678 (0.001) | 0.673 (0.003) |
| **14** | 1919  (6) | 1954  (21) | 2014  (3) | 1921  (12) | 0.668 (0.001) | 0.655 (0.003) | 0.67 (0.003) | 0.666 (0.001) |
| **15** | 1786  (3) | 1927  (23) | 1928  (19) | 1837  (14) | 0.704 (0.004) | 0.675 (0.006) | 0.702 (0.003) | 0.693 (0.002) |
| **16** | 1901  (11) | 1890  (18) | 1988  (14) | 1893  (14) | 0.695 (0.001) | 0.699 (0.003) | 0.693 (0.002) | 0.697 (0.004) |
| **17** | 1488  (6) | 1606  (15) | 1614  (21) | 1559  (5) | 0.643 (0.004) | 0.608 (0.003) | 0.627 (0.004) | 0.646 (0.003) |
| **Mean**  **(SD)** | 1953  (170) | 1986  (162) | 2072  (190) | 1961  (167) | 0.686 (0.027) | 0.674 (0.031) | 0.682 (0.028) | 0.683 (0.026) |

**Supplementary table 1a:** Mean |G*| (SD) in Pa and mean φ (SD) in rad for each phase and participant during the Valsalva experiment using the 42-Hz regimen (40.77, 41.67, 42.55 Hz). Baseline (BSL), established maneuver (ESM), late response maneuver (LRM), recovery (REC).

|  | Mean \|G*\| (SD) in Pa | | | | Mean φ (SD) in rad | | | |
| --- | --- | --- | --- | --- | --- | --- | --- | --- |
| **ID** | BSL | ESM | LRM | REC | BSL | ESM | LRM | REC |
| **1** | 1313 (5) | 1313 (7) | 1332 (1) | 1309 (13) | 0.8 (0.004) | 0.795 (0.002) | 0.797 (0.002) | 0.806 (0.001) |
| **2** | 1530 (7) | 1531 (14) | 1550 (2) | 1524 (3) | 0.78 (0.002) | 0.762 (0.005) | 0.77 (0.003) | 0.781 (0.004) |
| **3** | 1541 (7) | 1551 (12) | 1542 (3) | 1503 (2) | 0.801 (0.002) | 0.795 (0.002) | 0.794 (0.001) | 0.804 (0.002) |
| **4** | 1396 (12) | 1434 (10) | 1431 (4) | 1399 (12) | 0.767 (0.004) | 0.762 (0.003) | 0.765 (0.001) | 0.77 (0.002) |
| **5** | 1237 (9) | 1237 (2) | 1233 (2) | 1211 (4) | 0.806 (0.004) | 0.801 (0.001) | 0.808 (0.001) | 0.813 (0.003) |
| **6** | 1299 (10) | 1356 (13) | 1350 (1) | 1270 (10) | 0.756 (0.006) | 0.744 (0.002) | 0.75 (0.001) | 0.767 (0.003) |
| **7** | 1528 (3) | 1545 (5) | 1545 (1) | 1537 (4) | 0.794 (0.002) | 0.794 (0.001) | 0.794 (0.001) | 0.791 (0.001) |
| **8** | 1223 (10) | 1291 (12) | 1271 (6) | 1220 (8) | 0.815 (0.006) | 0.803 (0.001) | 0.812 (0.001) | 0.816 (0.001) |
| **9** | 1292 (8) | 1315 (3) | 1314 (1) | 1296 (7) | 0.844 (0.001) | 0.834 (0.002) | 0.85 (0.002) | 0.849 (0.001) |
| **10** | 1353 (7) | 1396 (26) | 1379 (4) | 1336 (5) | 0.792 (0.001) | 0.782 (0.002) | 0.791 (0.002) | 0.797 (0.004) |
| **11** | 1363 (8) | 1389 (9) | 1385 (1) | 1355 (3) | 0.789 (0.001) | 0.781 (0.003) | 0.784 (0) | 0.784 (0.001) |
| **12** | 1346 (11) | 1352 (13) | 1353 (10) | 1323 (4) | 0.773 (0.009) | 0.769 (0.001) | 0.778 (0.002) | 0.776 (0.003) |
| **13** | 1318 (5) | 1337 (9) | 1345 (4) | 1281 (6) | 0.822 (0.001) | 0.814 (0.001) | 0.82 (0.001) | 0.821 (0.002) |
| **14** | 1164 (5) | 1186 (7) | 1167 (5) | 1175 (3) | 0.73 (0.002) | 0.732 (0.004) | 0.732 (0.002) | 0.727 (0.003) |
| **15** | 1329 (6) | 1346 (6) | 1348 (3) | 1334 (9) | 0.781 (0.005) | 0.774 (0.003) | 0.769 (0.001) | 0.772 (0.001) |
| **16** | 1383 (5) | 1442 (7) | 1482 (3) | 1456 (7) | 0.831 (0.008) | 0.793 (0.001) | 0.822 (0.002) | 0.846 (0.002) |
| **17** | 1338 (3) | 1351 (6) | 1366 (3) | 1348 (3) | 0.844 (0.001) | 0.845 (0.002) | 0.83 (0.002) | 0.879 (0.001) |
| **Mean**  **(SD)** | 1350 (102) | 1375 (99) | 1376 (104) | 1345 (105) | 0.796 (0.03) | 0.787 (0.028) | 0.792 (0.029) | 0.8 (0.035) |

**Supplementary table 1b:** Mean |G*| (SD) in Pa and mean φ (SD) in rad for each phase and participant during the BH-in experiment using the 31-Hz regimen (30.03, 30.91, 31.8 Hz). Baseline (BSL), established maneuver (ESM), late response maneuver (LRM), recovery (REC).

|  | Mean \|G*\| (SD) in Pa | | | | Mean φ (SD) in rad | | | |
| --- | --- | --- | --- | --- | --- | --- | --- | --- |
| **ID** | BSL | ESM | LRM | REC | BSL | ESM | LRM | REC |
| **1** | 1306 (4) | 1302 (8) | 1317 (5) | 1313 (12) | 0.799 (0.003) | 0.799 (0.003) | 0.804 (0.003) | 0.821 (0.005) |
| **2** | 1536 (11) | 1541 (15) | 1568 (4) | 1538 (3) | 0.776 (0.003) | 0.773 (0.003) | 0.788 (0.003) | 0.779 (0.002) |
| **3** | 1520 (6) | 1533 (4) | 1522 (3) | 1506 (8) | 0.805 (0.003) | 0.803 (0.002) | 0.805 (0) | 0.798 (0.001) |
| **4** | 1386 (5) | 1422 (6) | 1436 (1) | 1428 (8) | 0.771 (0.003) | 0.757 (0.002) | 0.764 (0) | 0.758 (0.001) |
| **5** | 1234 (11) | 1251 (6) | 1249 (3) | 1227 (6) | 0.801 (0.004) | 0.796 (0.006) | 0.805 (0.001) | 0.806 (0.002) |
| **6** | 1292 (19) | 1327 (5) | 1322 (3) | 1277 (11) | 0.763 (0.007) | 0.759 (0.003) | 0.761 (0.002) | 0.771 (0.003) |
| **7** | 1542 (5) | 1557 (5) | 1572 (1) | 1561 (8) | 0.782 (0.003) | 0.785 (0.001) | 0.789 (0) | 0.785 (0.002) |
| **8** | 1232 (13) | 1300 (11) | 1302 (5) | 1229 (4) | 0.815 (0.006) | 0.804 (0.001) | 0.813 (0.002) | 0.815 (0.002) |
| **9** | 1293 (5) | 1331 (7) | 1344 (1) | 1307 (12) | 0.845 (0.001) | 0.844 (0.003) | 0.852 (0.001) | 0.842 (0.001) |
| **10** | 1362 (6) | 1381 (8) | 1359 (3) | 1356 (11) | 0.782 (0.001) | 0.794 (0.003) | 0.806 (0) | 0.781 (0.001) |
| **11** | 1360 (7) | 1373 (3) | 1366 (5) | 1368 (5) | 0.788 (0.001) | 0.79 (0.001) | 0.789 (0.001) | 0.778 (0.002) |
| **12** | 1364 (5) | 1345 (2) | 1366 (7) | 1338 (4) | 0.765 (0.005) | 0.774 (0.004) | 0.775 (0) | 0.771 (0.003) |
| **13** | 1326 (6) | 1336 (3) | 1318 (6) | 1287 (6) | 0.821 (0.002) | 0.824 (0) | 0.832 (0.001) | 0.82 (0.004) |
| **14** | 1153 (4) | 1153 (7) | 1161 (3) | 1165 (7) | 0.728 (0.002) | 0.722 (0.003) | 0.726 (0.001) | 0.725 (0.002) |
| **15** | 1338 (7) | 1342 (6) | 1357 (1) | 1325 (6) | 0.766 (0.003) | 0.77 (0.002) | 0.764 (0.001) | 0.768 (0.001) |
| **16** | 1537 (6) | 1533 (4) | 1561 (5) | 1559 (10) | 0.754 (0.007) | 0.761 (0.004) | 0.76 (0.002) | 0.77 (0.004) |
| **17** | 1235 (14) | 1302 (9) | 1312 (6) | 1244 (6) | 0.772 (0.005) | 0.784 (0,001) | 0.8 (0.004) | 0.775 (0.004) |
| **Mean**  **(SD)** | 1354 (115) | 1372 (109) | 1378 (113) | 1355 (119) | 0.784 (0.027) | 0.785 (0.027) | 0.79 (0.029) | 0.786 (0.027) |

**Supplementary table 1c:** Mean |G*| (SD) in Pa and mean φ (SD) in rad for each phase and participant in the BH-ex experiment using the 31-Hz regimen (30.03, 30.91, 31.8 Hz). Baseline (BSL), established maneuver (ESM), late response maneuver (LRM), recovery (REC).

Supplementary table 2 presents the number of CSF associated voxels for the individual phases and each participant for the VM using the 31-Hz regimen derived from automatic segmented CSF masks as shown in Figure 4 in the main manuscript.

|  | Number of CSF associated voxels | | | |
| --- | --- | --- | --- | --- |
| **ID** | BSL | ESM | LRM | REC |
| **1** | 940 | 889 | 892 | 876 |
| **2** | 1070 | 1205 | 1040 | 1106 |
| **3** | 1079 | 1128 | 1069 | 1074 |
| **4** | 1091 | 1019 | 939 | 1016 |
| **5** | 1078 | 860 | 1170 | 1141 |
| **6** | 1030 | 1028 | 1067 | 1102 |
| **7** | 1079 | 1129 | 855 | 1011 |
| **8** | 1284 | 935 | 1106 | 1236 |
| **9** | 1001 | 1064 | 907 | 1201 |
| **10** | 918 | 894 | 821 | 910 |
| **11** | 1037 | 1056 | 1023 | 982 |
| **12** | 889 | 1143 | 850 | 955 |
| **13** | 909 | 1204 | 1012 | 950 |
| **14** | 866 | 1112 | 944 | 1114 |
| **15** | 985 | 998 | 1151 | 1007 |
| **16** | 985 | 888 | 1065 | 1104 |
| **17** | 1178 | 1043 | 1198 | 1098 |
| **Mean**  **(SD)** | 1025  (108) | 1035  (112) | 1006  (117) | 1052  (100) |

Supplementary table 2: Number of CSF associated voxels derived from temporal averaged MRE magnitude images for each phase and participant during the Valsalva experiment using the 31-Hz regimen (30.03, 30.91, 31.8 Hz). Automatic segmentation was done using SPM12. CSF probability maps were thresholded at 0.5 to generate logical CSF masks. Group statistics using a linear-mixed model revealed no significant change of the CSF associated voxels between the different states of the maneuver. Baseline (BSL), established maneuver (ESM), late response maneuver (LRM), recovery (REC).
